# The nucleoid protein Dps binds genomic DNA of *Escherichia coli* in a non-random manner

**DOI:** 10.1371/journal.pone.0182800

**Published:** 2017-08-11

**Authors:** S. S. Antipov, M. N. Tutukina, E. V. Preobrazhenskaya, F. A. Kondrashov, M. V. Patrushev, S. V. Toshchakov, I. Dominova, U. S. Shvyreva, V. V. Vrublevskaya, O. S. Morenkov, N. A. Sukharicheva, V. V. Panyukov, O. N. Ozoline

**Affiliations:** 1 Department of Functional Genomics and Cellular Stress, Institute of Cell Biophysics of Russian Academy of Sciences, Pushchino, Moscow Region, Russian Federation; 2 Department of Cell Biology, Pushchino State Institute of Natural Sciences, Pushchino, Moscow Region, Russian Federation; 3 Department of Biophysics and Biotechnology, Voronezh State University, Voronezh, Russian Federation; 4 Department of Genomics of Microorganisms, Immanuel Kant Baltic Federal University, Kaliningrad, Russian Federation; 5 Bioinformatics and Genomics Programme, Centre for Genomic Regulation (CRG) Barcelona, Spain; 6 Department of Evolutionary Genomics, Universitat Pompeu Fabra (UPF), Barcelona, Spain; 7 Department of Structural and Functional Genomics,–Pushchino Research Center of the Russian Academy of Sciences, Pushchino, Moscow Region, Russian Federation; 8 Institució Catalana de Recerca i Estudis Avançats (ICREA), 23 Pg. Lluís Companys, Barcelona, Spain; 9 Department of Cell Culture and Cell Engeneering, Institute of Cell Biophysics of Russian Academy of Sciences, Pushchino, Moscow Region, Russian Federation; 10 Department of Bioinformatics, Institute of Mathematical Problems of Biology—the Branch of Keldysh Institute of Applied Mathematics of Russian Academy of Sciences, Pushchino, Moscow Region, Russian Federation; Hosei University, JAPAN

## Abstract

Dps is a multifunctional homododecameric protein that oxidizes Fe^2+^ ions accumulating them in the form of Fe_2_O_3_ within its protein cavity, interacts with DNA tightly condensing bacterial nucleoid upon starvation and performs some other functions. During the last two decades from discovery of this protein, its ferroxidase activity became rather well studied, but the mechanism of Dps interaction with DNA still remains enigmatic. The crucial role of lysine residues in the unstructured N-terminal tails led to the conventional point of view that Dps binds DNA without sequence or structural specificity. However, deletion of *dps* changed the profile of proteins in starved cells, SELEX screen revealed genomic regions preferentially bound *in vitro* and certain affinity of Dps for artificial branched molecules was detected by atomic force microscopy. Here we report a non-random distribution of Dps binding sites across the bacterial chromosome in exponentially growing cells and show their enrichment with inverted repeats prone to form secondary structures. We found that the Dps-bound regions overlap with sites occupied by other nucleoid proteins, and contain overrepresented motifs typical for their consensus sequences. Of the two types of genomic domains with extensive protein occupancy, which can be highly expressed or transcriptionally silent only those that are enriched with RNA polymerase molecules were preferentially occupied by Dps. In the *dps*-null mutant we, therefore, observed a differentially altered expression of several targeted genes and found suppressed transcription from the dps promoter. In most cases this can be explained by the relieved interference with Dps for nucleoid proteins exploiting sequence-specific modes of DNA binding. Thus, protecting bacterial cells from different stresses during exponential growth, Dps can modulate transcriptional integrity of the bacterial chromosome hampering RNA biosynthesis from some genes via competition with RNA polymerase or, vice versa, competing with inhibitors to activate transcription.

## Introduction

Dps was initially described as an abundant protein of starved cells (The **D**NA-binding **P**rotein from **S**tarved cells) [1] and later became a prototype for the Dps family of bacterial ferritins. Playing a key role in genome condensation [2], it is actively produced during stationary phase reaching 85,000–180,000 molecules per cell [3, 4], while exponentially growing cells of *Escherichia coli* (*E*. *coli*) possess only 6,000–8,500 monomers of Dps. Proteins from the Dps family are composed of 12 identical or similar subunits, and form spherical particles with an internal cavity of ~4.5 nm. In *E*. *coli* this protein is a homododecamer with 2–3 tetrahedral symmetry [5].

Dps is a multifunctional protein protecting bacterial cells from oxidative stress, UV- and γ-radiation, as well as metal ion toxicity [6–11]. Proteins of this family also play a role in biofilm formation [12], and were found among outer membrane proteins and fimbria [13]. The protective function is mostly conferred by DNA-binding and ferroxidase activities of Dps, which are topologically separated, but function jointly to preserve DNA integrity and cellular viability [14]. Operating as a ferritin, Dps oxidizes supertoxic but essential ferrous ions and accumulates them within its cavity in the form of iron oxides [15, 16]. Ferroxidase activity of Dps is relatively well studied, and it is not very different from other ferritins. Although Dps oxidizes Fe(II) predominantly using hydrogen peroxide, rather than oxygen, and 12 catalytic centers are formed by surfaces of the 2 adjacent subunits, rather than being located within the 4-helix bundle of each subunit [17].

An ability to bind and condense genomic DNA is a specific feature of proteins from the Dps-family [18]. Both inner [5] and outer [5, 19] surfaces of the *E*. *coli* Dps are charged negatively, thus it is not clear how this protein was evolutionary selected for interaction with negatively charged DNA. Based on the X-ray structure [5] and the work of Ceci *at al*. [18], it is thought that the N-terminal tails, lacking a DNA-binding module, but containing positively charged amino acid residues, are responsible for interaction with DNA, for Dps self-aggregation, and for binding/aggregation-mediated DNA condensation. Deletion of the first 8 amino acids including Lys5 and Lys8, or 18 amino acids with Lys10 and Arg18 decreased both self-aggregation and DNA condensation [18]. Nonetheless, the mutant Dps molecules still bound DNA as ‘beads on a string’, whereas Dps of *Listeria innocua* and two Dps-like proteins of *Bacillus anthracis* possessing short N-termini were not able to do this [17, 20, 21]. On the other hand, Dps from *Helicobacter pylori* (NAP), which does not have positively charged N-termini at all, binds DNA exploiting its positively charged protein surface [22]. The situation with the DNA-binding modules becomes even more ambiguous, if to take into account that the N-terminal tails of the two Dps proteins from *Lactococcus lactis* form alpha-helices potentially capable of binding to a large groove of DNA, but do not show any dependence on the presence of Lys residues in their structure [23].

*Mycobacterium smegmatis* genome also encodes two proteins of the Dps family [24, 25]. Of them, Dps1 is produced predominantly at stationary phase, whereas Dps2 is synthesized constitutively [26, 27]. Both these proteins participate in phase-dependent DNA packaging into toroid-like structures, but only toroids formed by Dps2 are further converted into more stable coral reef structures [28]. The mode of DNA packaging may, therefore, be different even for highly homologous proteins. In the *E*. *coli* cells highly packed Dps—DNA co-crystals were also registered at the late stationary phase. Their formation during steady growth was observed both with [29] and without [30–32] mild overproduction of Dps. However, in exponentially growing *E*. *coli* cells, no sign of crystallization has been detected, even in conditions of the plasmid-born Dps overproduction [30], or active production of endogenous Dps in response to oxidative stress [33]. Intracellular concentration of Dps is, therefore, not the only factor that is required to switch on protective heterochromatization of genomic DNA in *E*. *coli*. At the same time, the Dps orthologs from *Staphylococcus aureus* can transform nuleoid into a condensed state in both exponential and stationary phases [34]. Such a diversity of features and the lack of clear correlation between structural organization and functional properties make it challenging to understand the mechanism of Dps-DNA interaction.

The presence of functionally important [18] positively charged modules, suggests a simple electrostatic interaction between Dps and DNA without any sequence or structural specificity [1, 2, 18, 22, 30]. If so, deletion of *dps* would not have a large effect on the pattern of gene expression, and this was likely the case for the proteome of exponentially growing cell cultures [1]. However, the spectrum of proteins produced in bacteria during starvation showed dramatic and differential alterations [1]. These changes were explained by the lack of Dps-mediated global structural impact on the bacterial chromosome or by regulatory effects from transcription factors, occupying the sites occasionally bound by Dps and released in its absence. For all that, genomic SELEX revealed sequences that were preferably bound by Dps *in vitro* (data deposited in DataBase TEC https://shigen.nig.ac.jp/ecoli/tec/top/about [35]), and we detected an ability of Dps to choose one of the two competing DNA fragments for complex formation [19]. Dps, therefore, may have some sequence or structural selectivity. Some preference of Dps for the ends of linear DNA fragments and even higher affinity for the branching point of artificial 3-way junction molecules was detected by atomic force microscopy [19]. Based on these data, it was assumed that Dps tends to bind those sites in the genomic DNA, which have locally increased “concentration” of DNA providing a platform for the maximal number of N-termini. Given that N-termini are grouped in triades around ferritin-like vertices of the dodecamer, the 3-way junction anchoring the Dps particles at the branching point seemed to be an optimal target for interaction. However, bent DNA, Holliday junctions, or any other structural features, increasing the availability of DNA for N-termini may be attractive as well. Here, we checked if Dps has any structural or sequence preferences at the genome-wide scale.

ChIP-seq experiments performed for exponentially grown cells revealed 451 sites with affinity to Dps and about 1200 genomic regions remained unbound. The newly revealed targets significantly overlap with the Dps binding sites found by SELEX [35]. In comparison to the control unbound sequences, they appeared to be enriched with inverted repeats and apparently overlap with binding sites of several other proteins of the bacterial nucleoid. Biological significance of a combined or permutable interaction of several structural proteins within one and the same genomic region is discussed. We checked the possibility of Dps participation in transcription regulation, and differential changes in intracellular amount of five mRNAs, but not all, were detected in response to the *dps* gene deletion. In all cases apparently regulatory effects can be explained by relieved competition with Dps for nucleoid proteins recognizing specific sequence motifs in their targets. Thus, it became clear that Dps can “specifically” modulate transcription of at least those genes that are regulated by transcription factors binding their DNA-targets with a lower affinity than Dps.

## Materials and methods

### Bacterial strains

All experiments, except protein production described in the next paragraph, were done using *E*. *coli* K-12 MG1655 cells. The *dps*-null mutant was first made in *E*. *coli* BW25113 as described in [36] using primers dps_del_1 and dps_del_2 (S1 Table), and then the mutation was transferred to *E*. *coli* K12 MG1655 by P1 transduction as described in [37]. After removal of the kanamycin cassette with pCP20, the whole-genome sequencing (Illumina, MiSeq) was used to check the presence of required deletion and to exclude the *dps* transposition in some other place. Bacterial cells were grown aerobically in M9 medium supplemented with 0.2% glucose and 5% LB at 37°C under constant shaking (~120 rpm) in a water bath and harvested at OD ~0.6.

### Anti-Dps antibodies

The Dps protein was purified to homogeneity as described in [19]. In brief, the *E*. *coli dps* gene was amplified with primers dps_cl_1 and dps_cl_2 (S1 Table) and cloned into the plasmid pGEMΔXba [38] using the Xba I site. No tag was added to the recombinant gene. The nucleotide sequence of the insert was checked by direct sequencing. The gene was expressed in *E*. *coli* BL21(DE3) cells grown in LB Medium in the presence of ampicillin (100 μg/mL). Its transcription was induced by 0.02 mM IPTG at OD600 0.4–0.6, and biosynthesis of the protein was allowed for 12 h. Dps was purified using ion exchange chromatography on DEAE-Sephadex A-25 (GE Healthcare, Sweden) and gel-filtration on Sephadex G-200 (Pharmacia, Sweden). The protein was dialyzed against the buffer containing 50 mM Tris-HCl (pH 8.0), 50 mM NaCl, 0.1 mM EDTA and concentrated on Amicon Millipore 3kDa (Merck, USA) spin columns. The final purity of the native protein was higher than 95%. Two New Zealand rabbits were immunized subcutaneously with 100 μg of Dps emulsified in incomplete Freund’s adjuvant. Three booster injections were given using 75 μg of Dps emulsified in the same adjuvant with an interval of two weeks between injections. Animal health was daily monitored to ensure their healthy state at the end of experiment. Blood, collected from the rabbits 14 days after the last immunization, was used to prepare antisera. Antibodies were purified from the antisera by ion exchange chromatography on DEAE-Toyopearl as described in [39]. Final concentration of anti-Dps IgG determined with spectrophotometer ND-1000 (NanoDrop Technologies Inc., USA) was 3mg/ml. Antibodies were precipitated by 60% ammonium sulfate and stored at 4°C until use. All works with animals were carried out in accordance with the “Guidelines for Biomedical Research Using Animals” accepted in the Institute of Cell Biophysics 30.12.2011 and have been approved by the Animal Care and Use Committee of the Institute of Cell Biophysics of Russian Academy of Sciences. At the end of the experiment, the rabbits were euthanized with CO_2_ inhalation according to the GLP standards for euthanasia using carbon dioxide.

### ChIP-seq experiments

ChIP-seq experiments were performed in duplicate using similar growth condition and protocols for chromatin immunoprecipitation. The first experiment was done in the Centre for Genomic Regulation (CRG, Barcelona, Spain), while the second one in the Immanuel Kant Baltic Federal University (Kaliningrad, Russia). In both cases, the chromatin was cross-linked, isolated and precipitated basically as suggested in [40] with certain modifications. Bacterial cells were grown aerobically at 37°C until OD_600_~0.6, then formaldehyde was added to a final concentration of 1% and incubation was allowed for 20 minutes. Cross-linking was stopped with glycine (final concentration of 450 mM). After 5 minutes of incubation, the cells were pelleted by centrifugation at 14,000 rpm for 15 minutes (+4°C), washed twice with 5 ml of PBS and resuspended in 1.3 ml of ice-cold immunoprecipitation buffer prepared from 50 ml of buffer containing 100 mM NaCl, 50 mM Tris-HCl (pH 8.1), 5 mM EDTA, 0.2% NaN_3_, 0.5% SDS, and 25 ml of buffer containing 100 mM Tris-HCl (pH 8.6); 100 mM NaCl; 5 mM EDTA; 0.2% NaN_3_, 5% Triton-X-100. Then phenylmethylsulfonyl fluoride (final concentration of 1 mM) or 20 μl of Protease Inhibitor Cocktail (PIC, Sigma) for the first and the second experiment, respectively, were added followed by incubation for 30 min at +4°C.

To shear the chromatin, in the first experiment samples were subjected to 18 cycles of sonication on Bioruptor (Diagenode, USA). In the second experiment, 40 cycles of sonication on 150 Soniprep Plus (MSE, UK) were applied. Cell debris was removed by centrifugation (15 min at 14,000 rpm and 4°C). Supernatant was checked for shearing quality and used for further procedures. The size of the obtained DNA fragments was in the range of 150–300 bp and 300–500 bp for the first and the second experiment, respectively.

For immunoprecipitation, 800 μl of collected chromatin (~1000 μg of protein) were incubated on a rotating wheel over night at 4°C with 10 μg of either rabbit anti-Dps antibodies (experimental sample) or rabbit pre-immune IgG (negative control). Next day 30 μl of Ultra Link Protein A/G beads (Thermo Scientific, USA) were added to the samples, and incubation was allowed for a further 2 hours at 4°C on a rotating wheel. The beads were washed 3 times with 1 ml of the low-salt buffer (50 mM HEPES pH 7.5, 140 mM NaCl, 1% Triton X-100, 1xPIC) and then with 1 ml of the high-salt buffer (500 mM NaCl). Immunoprecipitated DNA-protein complexes were removed from the beads by 3 h shaking at 65°C and 1000 rpm in 110 μl of freshly prepared elution buffer, containing 100 mM NaHCO_3_ and 1% SDS. After centrifugation at 3000 rpm for 5 minutes, 100 μl of the supernatant was transferred to a new tube, and the DNA was purified with a PCR Purification Kit (Qiagen, Germany). The DNA concentration was measured on Qubit 2.0 using Qubit dsDNA HS Assay kit. A total of 8–10 identical samples were combined and concentrated to a volume of 30–50 μl containing 5–10 ng of the DNA. Before library preparation, the samples were qPCR-checked for the enrichment with the target DNA. Assuming that Dps can bind the regulatory region of its own gene, two fragments from the *dps* regulatory region amplified with primer pairs dps_F1 –dps_R1 and dps_F2 –dps_R2 (S1 Table) were used for validation.

### Library preparation

ChIP-seq libraries were prepared from 5–10 ng of the DNA samples with the NebNext Ultra DNA Library Prep Kit for Illumina (New England Biolabs, MA, USA) following the manufacturer’s instructions. For the final amplification of the library 15 PCR cycles were used. Size distribution and concentration of the amplicons was checked on the Bioanalyzer 2100 (Agilent, USA). In the first experiment, the maximum was at about 300 bp, and ChIP libraries were sequenced using 50 nt single-end read protocol on the Illumina HiSeq system (Illumina 2000, USA) of the Genomics Facility in the Centre for Genomic Regulation (Barcelona). In the second experiment, the maximum was at about 450 bp, and samples were sequenced using standard paired-end 2*150 nt protocol on the MiSeq system (Illumina, USA) in the Immanuel Kant Baltic Federal University (Kaliningrad).

### Chip-seq data evaluation

Raw data were quality checked with the FastQC software (http://www.bioinformatics.babraham.ac.uk/projects/fastqc/). The output files obtained from Illumina HiSeq contained 32,860,548 and 47,147,298 of 50 bp single-end reads in the control and immunoprecipitated samples, respectively. The output files from Illumina MiSeq contained, respectively, 10,028,552 and 11,570,890 reads, varying in the range from 35 to 150 nucleotides. All four sets were quality filtered on the Galaxy server (Filter by Quality tool, Q≥20) providing 99% probability of correct sequencing for all nucleotides in each read [41]. As a result, our sets contained 31,656,551 and 45,396,252 sequences in the control and immunoprecipitated samples of the first experiment, and, respectively, 7,493,528 and 8,214,737 reads for the second experiment. The data discussed in this publication have been deposited in NCBI's Gene Expression Omnibus [42] and are accessible through GEO Series accession number GSE102091 (https://www.ncbi.nlm.nih.gov/geo/query/acc.cgi?acc=GSE102091).

Two different approaches were used to align sequence reads to the genome of *E*. *coli* K-12 MG1655 (U00096.3). First, quality controlled reads were mapped using the CLC Genomics Workbench version 7.5.1 (CLC GW, Bio-Qiagen, Aarhus, Denmark) with either default settings for the length and similarity fractions (0.5 and 0.8, respectively) or with the most stringent criteria (1.0 and 1.0, respectively). Reads with sequences aligned to multiple genomic regions were ignored. In both cases, peak calling provided as much as several thousand of the Dps-bound areas with p-value less than e-3.

To apply the second approach variable in length reads from Illumina MiSeq were first trimmed from both sides to obtain a set of standard 50 nt sequences taken from the middle of longer reads. Reads shorter than 50 nt were discarded. Then, all four sets from both experiments were aligned to the genome using the Matcher program [43] (available at: http://www.mathcell.ru/DnaRnaTools/Matcher.zip). This software maps only 5’-ends if the reads correspond to the top strand of genomic DNA, or only 3’-ends, if they are aligned to the bottom strand. As such, the signals from fully complementary reads will match the same (left) position. Reporting the distribution of matching reads across the genome the program also evaluates reads with multiple occurrence and their positions. Only ideal correspondence to the genome was permitted. The profiles obtained for experimental and control samples were normalized by the scaling method initially offered by Affymetrix for microarray data analysis [44] and later implemented in several other approaches [45]. This method assumes unaffected protein occupancy at most genomic positions and quantifies the scaling factor on the basis of corrected mean values obtained after removal of 2% signals with highest and lowest intensities from both control and experimental sets.

After normalization, the read counts were estimated in the running windows of 25, 35 or 75 bp, and the ratios **R** between values obtained for experimental and control libraries were calculated. Peaks were localized requiring **R**≥1.5 for at least 50% positions of at least 60 bp genomic region in length (corresponds to the assumed size of the Dps binding site [18]). Genomic regions with **R**<1.0 for all positions in both experiments, spanning for at least 60 bp that did not overlap with peaks found by CLC GW with p-values < 1e-4 were collected into the set “unbound regions” (UR). Regions separated by less than 30 bp were combined if **R**-values in all intermediate positions did not exceed 1.1.

### Search for genomic units that overlap the Dps binding sites

More than a hundred sets of genomic regions with different functional loads were compared with the selected set of the Dps binding sites. These genomic units included binding sites of different proteins obtained by ChIP-chip [46–48] or ChIP-seq [49–51], *promoter islands* found computationally [43, 52], REP-elements annotated in the *E*. *coli* genome, and direct or inverted repeats identified by the Unipro UGENE software package [53].

Before analysis genomic coordinates of all data sets were translated into current version (U00096.3) of the *E*. *coli* MG1655 genome. Then, the total areas in the genome occupied by Dps and each protein of interest or a specific element were estimated. Thereafter, an expected number of overlapping base pairs in the two analyzed sets, in the case of their independent distribution, was calculated and compared with the observed intersection. All used ChIP-chip and ChIP-seq data were processed by their authors. These data, in which protein binding sites are listed with left and right borders [48–51], were directly used for positional comparison. In ChIP-chip data sets [46, 47] the lists of 60 bp microarray probes with averaged and normalized enrichment ratio were provided. Thus, the probes with at least 2-fold enrichment were selected, and 60 bp regions were assessed for overlap with genomic regions bound and unbound by Dps. An overlap with RNAP σ^70^ binding sites (aerobic growth) obtained by ChIP-seq and presented as point coordinates of peak maxima for 36 bp reads [50] was evaluated by the same way.

### Search for dominant sequence motifs

Nucleotide sequences over presented within the Dps binding sites were searched by the MEME suite programs (version 4.11.2, http://meme-suite.org/tools/meme, [54]) and DMINDA (http://csbl.bmb.uga.edu/DMINDA/annotate.php#tabs-1, [55]) as described in the text. Both software packages were run in the discriminative mode so as to discover motifs enriched in the experimental library compared to the set of sequences that are not bound by Dps.

### qRT-PCR

The total fraction of cellular RNAs was isolated from the wild type *E*. *coli* K12 MG1655 and the *dps*-null mutant cultures using TRIzol reagent (Ambion, USA) and then treated with DNAse I (New England Biolabs) following the manufacturers’ protocols. Primers used are listed in S1 Table. cDNAs were obtained in 25 μl of the reaction mixture, containing 1 or 2 μg of RNA and 80U of RevertAid reverse transcriptase (Thermo Scientific, Lithuania) according to the manufacturer’s protocol. Two microliters of this mixture were subsequently used for PCR performed in 20 μl with 4 μl of qPCRmix-HS SYBR (Evrogen, Russia). qPCR was run on DT*lite* thermal cycler (DNA Technology, Russia). The PCR program included 1 minute of initial melting at 95°C, followed by 35 cycles: 95°C for 20 s, annealing at 56°C for 20 s, and synthesis at 72°C for 25 s. The fluorescence of SYBR Green I was measured at the end of each cycle for 15 s. Samples without reverse transcriptase were used as negative controls. Two RNA products were used as a reference to estimate expression efficiency of chromosomal genes. The first one, transcribed in antisense direction to *ysaA*, was used previously [56]. The second one (*lacZ*-mRNA) was selected from the set of regions unbound by Dps (page 4 in S2 Table). The data obtained with two reference products were averaged. In qRT-PCR experiments for GFP-reporter assays (see below), mRNA of kanamycin resistance gene (*kan*) was used as a reference product. Specificity of the synthesized products in all cases was confirmed by electrophoresis in 5% polyacrylamide gel. The threshold cycles calculated by the qPCR software (DNA Technology, Russia) for the target and the reference genes in both the wild type and the *dps*-null mutant samples were used to estimate the mutation-mediated changes. Relative abundance was calculated using 2^-ΔΔ*CT*^ method. The computed values were expressed as fold ratios and averaged between 4–8 biological samples with 2, 3 or 6 technical replicates for each of them. Statistical significance of differences was assessed using the Student's t*-*test.

### GFP-reporter assays

To assess the ability of Dps to affect expression of its own gene, the *dps* regulatory region (primers dps_F1 and dps_R2) was cloned upstream of the reporter *gfp* gene in the plasmid pET28b-eGFP [57]. It was then transformed into the wild-type and the *dps*-null mutant cells. Fluorescence intensity was registered in colonies grown on LB agar supplemented with kanamycin (20 μg/ml) using a Leica microscope (2.5x objective). Plasmid with no promoters inserted upstream the *dps* gene was used as a control. Data were processed with ImageJ software [58].

## Results

### Dps binds genomic DNA in a non-random manner

Two data sets from the ChIP-seq experiments were obtained on HiSeq 2000 and MiSeq Illumina systems, providing sequence libraries with a substantially different sequencing depth. From the first experiment, 27,850,801 reads of the control set and 42,133,025 reads of immunoprecipitated sample were aligned to the *E*. *coli* MG1655 genome, with default settings in CLC GW. Stringent criteria gave 25,682,627 and 39,199,442 mapped reads, respectively. In the MiSeq experiment, we obtained 5,952,682 and 7,102,289 aligned reads with the default settings of CLC GW, while stringent criteria gave 4,341,979 and 5,788,335 hits, respectively. Depending on the settings, 14,875 or 14,309 peaks at least 60 bp in length were found in the first experiment with average p-values 5.9E-03 or 6.4E-03. If the peaks located at a 0–90 bp distance were combined (fused peaks are shaded gray in Pages 1 and 2 of S2 Table), the number of targeted areas decreased, but was still large (10,516 and 10,241 peaks, respectively). The second experiment gave, respectively, 5,350 and 4,772 combined peaks (Pages 3 and 4 of S2 Table) with average p-values 7.5E-03 and 9.2E-03. About 85% of the peaks detected in the second experiment (both settings) overlap those from the first experiment (the overlapping regions are marked bold in S2 Table). Given that the experiments were done in different laboratories and the samples were sequenced on different platforms, this similarity can be considered good, providing a chance to detect the core set of preferred Dps binding sites in the genome.

To validate them, we analyzed the bar chart representing the genome-wide profile of Dps targets detected in a SELEX experiments *in vitro* (TEC DataBase (https://shigen.nig.ac.jp/ecoli/tec/top/, [35]). From these data 371 peaks were localized with 100 bp accuracy, of which 326 overlapped with the peaks revealed in one or both our experiments. Thus, the data obtained *in vivo* (S2 Table) and *in vitro* (https://shigen.nig.ac.jp/ecoli/tec/top/) assume the presence of preferred targets for Dps in the genome of *E*. *coli*.

Nevertheless, before a detailed analysis of the received output data, at least two issues should be addressed. First, based on the automatic peak calling, an average size of the sites occupied by Dps in the two experiments appeared to be different: 102–104 bp and 214–220 bp (S2 Table). This discrepancy could reflect some functional features that were differentially realized in the two experiments or arise due to different length of sequence reads that have been mapped and subjected to peak calling (standard 50 nt in the first experiment and 35–150 nt in the second). For that reason, the next screen was made for 50 nt sequences only. In the case of the second experiment they were taken from the centre of longer reads.

**Fig 1:**
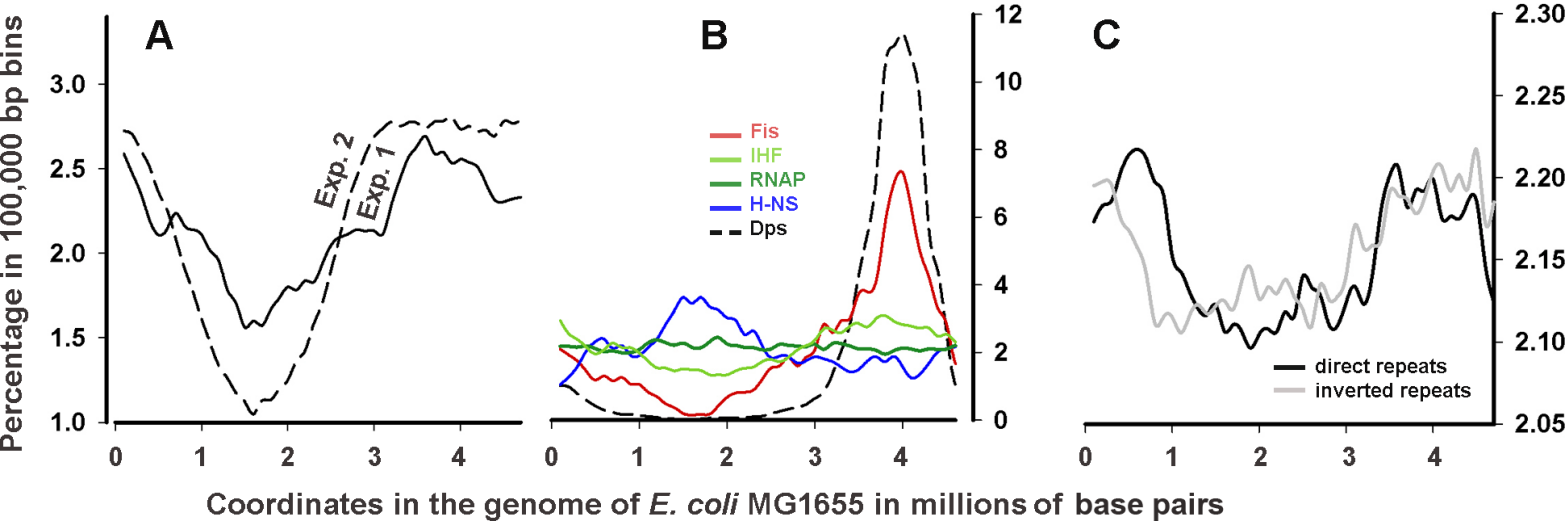
Large-scale profiles of the Dps targets correlate with the landscape of direct and inverted repeats and the pattern of Fis binding sites. **A:** Distribution of the Dps contact regions along the *E*. *coli* MG1655 genome identified by CLC GW in two experiments (the default settings). The areas covered by Dps are combined in 100,000 bp bins and plotted as percentage to the total length of all sites occupied by Dps. **B**: The same for the contact sites of Fis [49], IHF [51], H-NS [49] and RNAP [50] from the cells grown in conditions similar to those used in our experiments. The plot for Dps shows the distribution of the sites from the combined set (**CS**) (Page 3, S3 Table). **C:** The same for direct (5–24 bp separated by 1–15 bp) and inverted (5–18 bp separated by 3–20 bp) repeats collected from the genome of *E*. *coli* MG1655 using Unipro UGENE [53].

**Fig 2:**
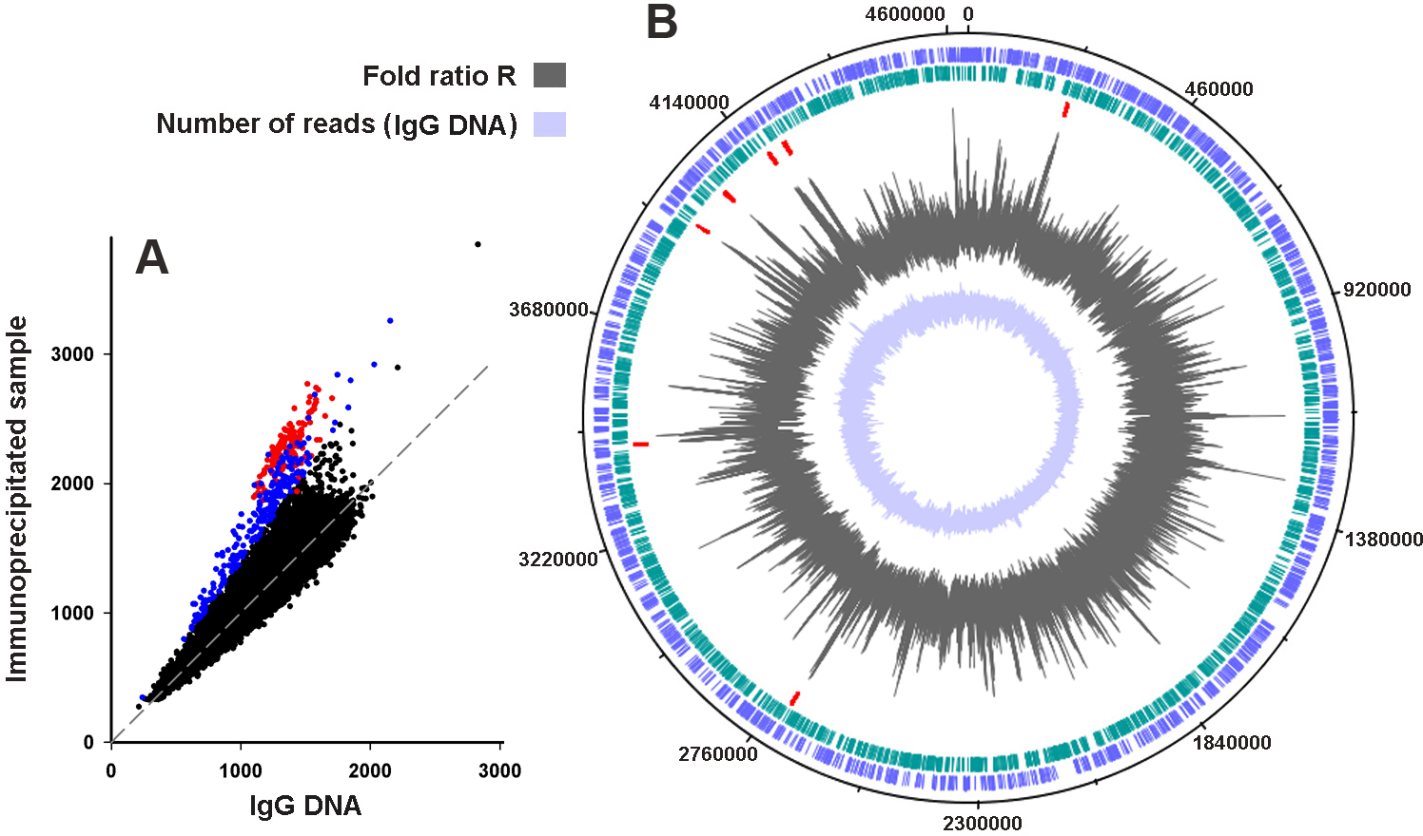
Dps binds genomic DNA in a non-random manner. **A:** Correlation between the number of reads from immunoprecipitated and control sequence libraries calculated for 100 bp bins (first experiment). All points, corresponding to rRNAs operons are marked in red. All other points with **R**-values higher than 1.4 are plotted in blue. Dashed line shows the bisectrix of the plot. **B:** Distribution of the Dps-binding sites in the genome. Two outer circles represent the gene map of the top and the bottom strands of the *E*. *coli* MG1655 genome. The red ticks on the third circle mark positions of rRNA operons. The profile of **R**-values and the distribution of reads registered in the control library calculated for a 35 bp running window are plotted on the fourth and fifth circles, respectively.

Second, both large-scale distributions of revealed Dps-binding sites show a similar bias towards the first half of replicores (Fig 1A). This bias may indicate specific enrichment of this region with the preferred Dps targets or, otherwise, reflect the natural difference in the DNA copy number that is higher near the origin of replication (Figs 2B and S1 central circles, [59]). CLC GW relies on the peak shape scores, which are calculated for the complete genome and identifies peaks as genomic regions where these scores are greater than a given threshold. To compensate the copy-number mediated bias, the peak calling in the next screen has been done using the **R** parameter estimated as a fold ratio between the normalized number of sequence reads registered in the immunoprecipitated and control sequence libraries.

The reads from both experiments were aligned to the genome using Matcher program [43] and analyzed as described in Materials and Methods. Correlation plots for control and immunoprecipitated samples are shown in Figs 2A and S1A. They demonstrate a high quality of normalization for both experiments and also indicate the presence of outliers. Many windows with **R**-ratio ≥ 1.4, found in the first experiment (blue dots in Fig 2A) were also found among outliers of the second experiment (blue symbols in S1A Fig), but the preferential interaction with ribosomal operons was detected only in the first one (red dots in Figs 2A and S1A). Thus, providing a chance to identify a common set of regions bound by Dps in both experiments, the data also revealed certain rearrangements in their profiles, which were caused by some untraceable differences in growth conditions.

Peak calling was made using the **R**-ratio of read counts found in 25, 35 or 75 bp running windows in the libraries of immunoprecipitated and control samples aligned to the genome. Two shorter windows gave contact sites better corresponding to each other and to the peaks found by CLC GW (see Figs 2B and S1B for 35 and 25 bp running windows, respectively). Of them 35 bp window also decreased the number of “empty” areas. It was, therefore, used to obtain Dps binding sites for further analysis.

Only 193 binding sites for Dps were found in the first library (Page 1, S3 Table), including seven very long regions (over 5,000 bp) with genes of ribosomal RNAs (red outliers in Fig 2A) and many tRNAs genes (Page 1, S3 Table). These genomic regions were poorly presented or absent in the set obtained by CLC GW, since reads with multiple matching to the genome were ignored. Yet, 178 out of 193 (92%) sites, found by the Matcher-based approach, at least partially overlap with peaks collected by CLC GW (indicated in bold in Page 1, S3 Table). Approximately 50-fold difference in the number of peaks revealed by the two approaches (Pages 1–2, S2 Table and Page 1, S3 Table) was not too surprising. Searching for peaks exceeding threshold for a given number of sequence reads, CLC GW can find more regions with high shape scores than Matcher does requiring higher than given cut off level fold-ratio between control and experimental samples. The values of R-ratio in the selected peaks of the first experiment varied from 1.52 to 2.56, thus the scale of fold changes was rather narrow and roughly similar across the genome (Fig 2B). Dps targets found by the Matcher-based approach appeared to be longer than those found by CLC GW (average size even without rRNA operons equals to 292 bp). Though, the size distribution histogram has a maximum in the range of 110–130 bp (S2B Fig) that nicely corresponds to the values detected by the CLC GW-mediated screening (100–110 bp, S2A Fig).

In the second experiment, Matcher revealed 1647 Dps binding sites (Page 2, S2 Table, S1B Fig). This is only three times fewer than in the case of the CLC GW screening, mainly because of lower coverage and, correspondingly, a lower background as compared to the first experiment. The range of the R-value variations in the peak maxima in this case was slightly wider: from 1.67 to 9.63, but still narrower, than it is typical for the binding sites of transcription factors (see, for instance ref. [47] and [50] for FNR). Again, a large part of sites, found by the Matcher-based mapping (83%), at least partly overlapped with the peaks collected by CLC GW (indicated in bold in Page 2, S3 Table). An average length of the Dps binding sites estimated on the basis of the second screen (195 bp, Page 2 in S3 Table) was approximately the same as assessed previously (214–220 bp, Pages 3 and 4 in S2 Table). However, the peak maxima in the size distribution histogram that was in the range of 160–190 bp (S1A Fig), shifted to 110–130 bp, coinciding with the first experiment (S1B Fig). As such, eleven-thirteen DNA helix turns can be suggested as a tentative estimate for the size of the site occupied by Dps *in vivo*, although it is twice as long as the estimate made *in vitro* on the basis of titration assays [18].

The set of the Dps targets detected in both experiments contains 451 genomic regions (Page 3, S3 Table). It was composed on the basis of 1647 peaks found in the second experiment (Page 2, S3 Table), from which the sites that were unbound in the first experiment were removed. Although the bias conditioned by an increased DNA copy number near the replication origin was, at least partially, compensated by the **R**-ratio, the binding sites detected in both experiments were mainly found in the last quarter of the genome (Fig 1B, dashed line). The most stable pattern of interaction is, therefore, located in this region. These genomic regions are further referred as CS (combined set). They were compared with 1227 genomic loci unbound by Dps in both ChIP-seq experiments and SELEX screening (Page 3, S3 Table), hereinafter referred as UR.

### Dps-binding sites are enriched with inverted repeats

Using electrophoretic mobility shift assays we recently found that *in vitro* Dps effectively binds artificial DNA molecules with a 3-way junction, and the direct contact with these polynucleotides was observed in their branching point [19]. Thus, we assumed that stronger binding may take place at the DNA loci that provide a platform for the simultaneous interaction with several N-tails of the Dps particle due to locally increased DNA concentration. If so, it might be expected that the Dps binding sites are enriched with inverted and/or direct repeats that can form helix-loop structures in the negatively supercoiled DNA due to alternative interactions with neighboring complementary sequences on the same or opposite strand.

Genomic distribution of 5–24 bp-long direct repeats separated by 0–20 bp, and 5–18 bp inverted repeats separated by 3–20 bp was obtained with the integrated Unipro UGENE toolkit (http://ugene.net/) [53, 60]. To estimate the difference between the expected and the observed overlap of these ubiquitous genomic elements with the Dps binding sites, each pair of repeats was considered as a holistic unit, and the total intersection of each type of repeats with the combined set of Dps targets (Page 3, S3 Table) was evaluated with a 1 bp resolution.

**Fig 3:**
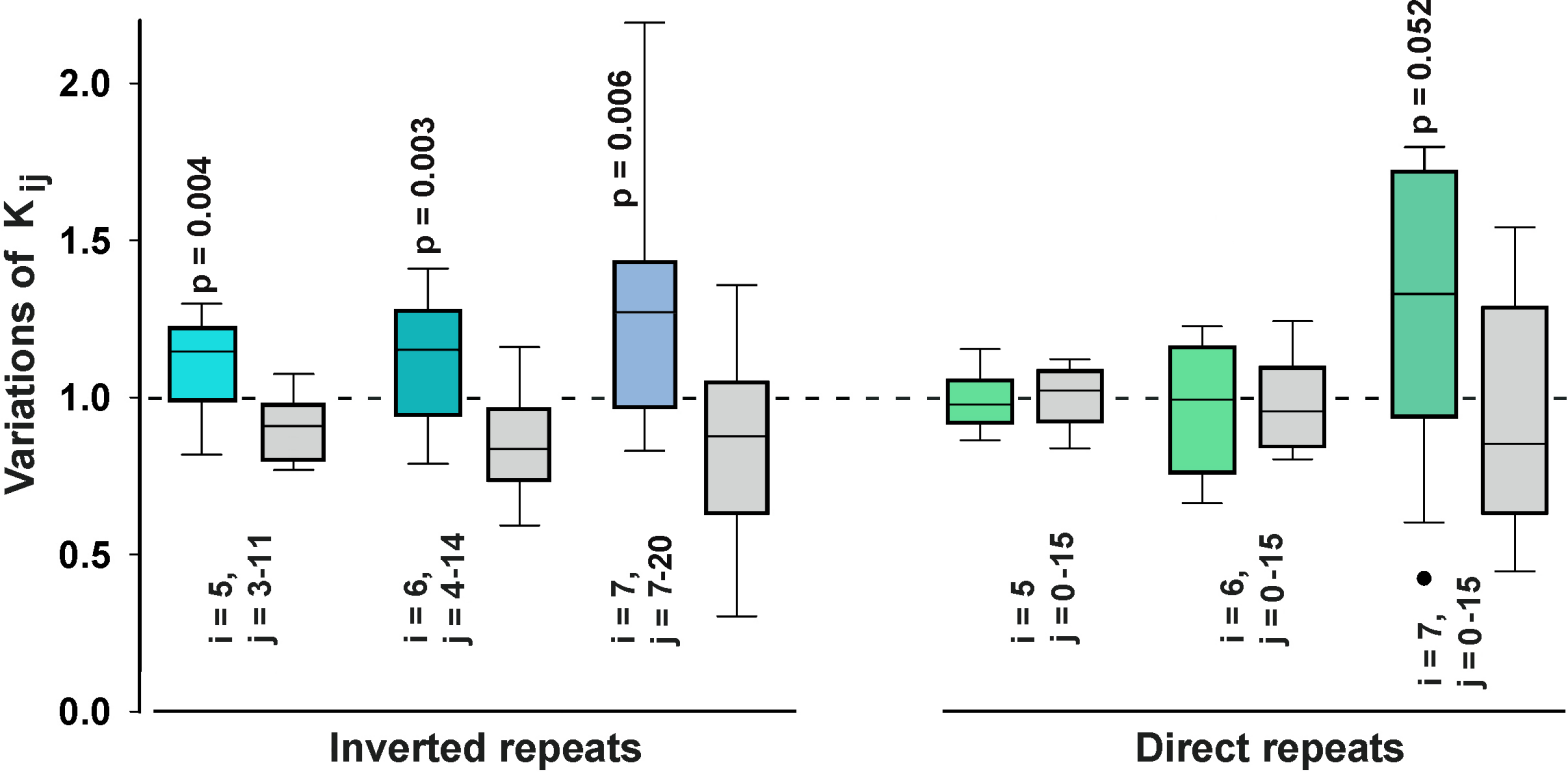
Dps-binding sites are enriched with inverted repeats. The overlap between sequences of CS (colored box-plots) and UR (gray boxes) with direct or inverted repeats was characterized by the parameter **K**_**ij**_ as described in the text. Black dot on the right panel shows one outlier. Box-plots with statistically significant differences are provided with corresponding p-values. Regions of bound and unbound sets overlapping with repeated sequences of both types are indicated in S3 Table.

The number of overlapping nucleotides was then compared with the value expected by chance and the ratio between these two numbers (**K**_**ij**_) was used as a measure of deviation from the expected co-occurrences for pairs of different lengths (**i**) and spacers (**j**). For instance, the bacterial genome contains 2993 inverted pentanucleotides separated by 3 bp. Their total length in the genome is 38909 bp (0.838%), assuming 916 bp overlap with 109330 bp of CS. In reality we found 1134 common base pairs, which gave 1.24-fold excess over expected ratio (**K**_**i-5,j-3**_ = 1) and one (i = 5, j = 3) of nine values (i = 5, j = 3–11) to the first box-plot in Fig 3. Using this approach, we estimated **K**_**ij**_ values for all pairs of repeats, but only those that were 5–7 bp long gave statistically reliable values (Fig 3) without zero occurrences in both sets.

We found that pairs of inverted repeats tend to be overrepresented at the Dps binding sites and underrepresented in the set of unbound regions. The statistical significance of the difference was maximal in the ranges of distances (**j**) shown in Fig 3. Thus, we conclude that Dps-binding sites are enriched with inverted repeats, which may facilitate or stabilize the Dps-DNA interaction. The situation with direct repeats, which large-scale distribution in the genome follows the profile of inverted repeats (Fig 1C), is less clear. The numbers of penta- and hexanucleotides in CS and UR vary in approximately the same range, and **K**_**ij**_ are close to the expected values (Fig 3). The number of heptanucleotides within Dps binding sites tends to be higher than in UR, but the difference is statistically insignificant (p = 0.056).

### Dps has high affinity to REP-elements and *promoter islands*

**Fig 4:**
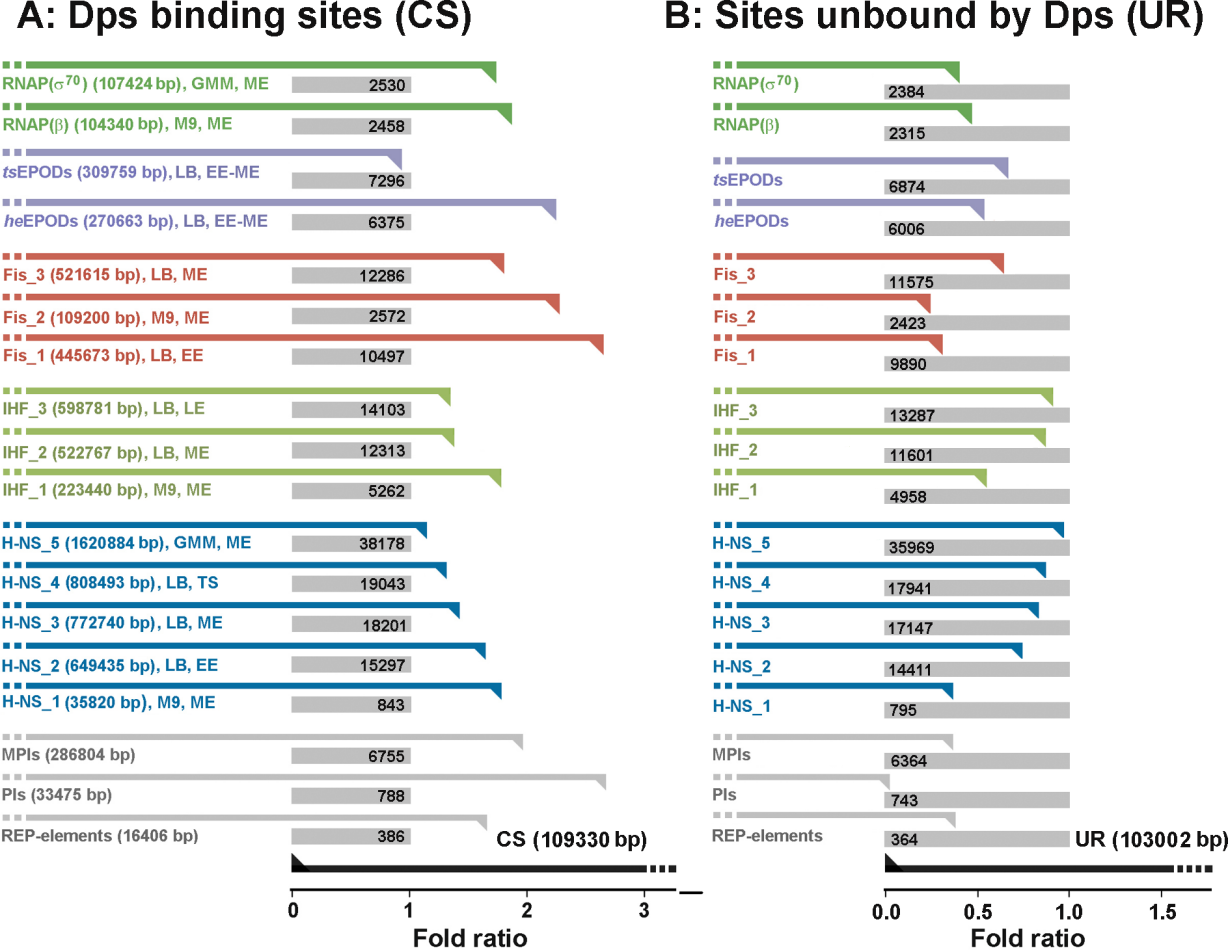
Dps shares its binding sites with other proteins of bacterial nucleoid and has affinity to REP-elements and *promoter islands*. Intersection of Dps targets (**A**) and sites unbound by Dps (**B**) with structural and functional elements of bacterial genome was estimated as described above for repeated sequences and plotted as fold ratio to the expected values. Bent black arrows on the bottom schematically show areas occupied by CS or UR. Gray rectangles and numerals inside indicate the expected number of common base pairs if compared modules are independently distributed along the genome. Gray and colored bent arrows show registered overlap calculated in 1 bp resolution. Numerals in parenthesis indicate the size of compared sets. Genomic locations of REP elements were taken from KEGG DataBase (http://www.genome.jp/kegg/, [63]), and fold ratios obtained for 302 REP-sequences containing 1–3 REP-modules (14–100 bp) were plotted. Analyzed ChIP-chip and ChIP-seq data sets were obtained from [46–51] for cells grown in LB medium (LB), M9 medium with fructose (M9) or MOPS minimal medium with glucose (GMM), harvested at early (EE), middle (ME) or late (LE) exponential phase or upon transition to the steady growth (TS).

The *E*. *coli* genome has two types of structure-specific elements with repeated sequences. They include interspersed across the genome REP-elements (**R**epetitive **E**xtragenic **P**alindromic sequences) [61] and *promoter islands* [62]. REP-elements are 25–35 bp in length and usually occur in multiple copies at one genomic location [61]. Nearby copies are always in inverted orientations potentiating their folding in complex secondary structures with some participation of short palindromes present in a single copy. We analyzed the overlap of CS and UR with all 355 REP-sequences annotated in the genome of *E*. *coli* [63], and found a 1.65-fold excess in the CS intersection level with 302 REP-elements containing 1–3 copies of the repeating sequences (Fig 4A). Their occurrence in the UR set, on the contrary, was significantly below normal (Fig 4B). Short REP-elements can thus be considered as suitable targets for interaction with Dps.

*Promoter islands* have a high density of closely located or even overlapping promoters in both orientations [43, 52] creating repeats of both type. Two sets of *islands* were used for comparison. The first one, PIs, contained 78 promoter-rich regions initially found by σ^70^-specific promoter finder PlatProm [52]. Another one was composed of 434 *mixed promoter islands* (MPIs) found by the unified version of PlatProm searching for promoter-like signals on the basis of structural information, which is similar for promoters of different σ-factors [43]. Both sets of *promoter islands* significantly overlapped with the sites occupied by Dps (Fig 4A), while their presence in the UR set was much lower than expected by chance (Fig 4B). Dps, therefore, has enhanced affinity for *promoter islands*.

### Dps shares binding sites with structural proteins of bacterial nucleoid

The interest to *promoter islands* was stipulated by their specific structure and association with horizontally acquired genes [43, 64]. Such genes are usually subjected to silencing by H-NS [65, 66] that occupies up to 90% of PIs during exponential growth [64]. As estimated above, about 13% of the *islands* were in contact with Dps (Page 3, S3 Table), allowing a potentiality for simultaneous interaction with these two proteins. Of 57 Dps binding sites, overlapping with *promoter islands*, 54 can also bind H-NS (Page 3, S3 Table). In CS, there are 181 Dps-bound sequences targeted by H-NS (Page 3, S3 Table). Maximal excess (1.76-fold) was obtained for cells grown in M9 media and harvested at exponential phase (Fig 4A, [46]), but the overlap of Dps and H-NS binding sites is likely to decrease upon transition to a steady growth (Fig 4A). The intersection of CS with IHF was approximately the same (Fig 4A). It was found for 177 Dps targets, of which 91 can also interact with H-NS (Page 3, S3 Table). The maximal overlap of CS was detected for Fis binding sites (1.8–2.6 fold excess over expected value). That was anticipated, as Fis and Dps are the most abundant nucleoid proteins in bacterial cell during exponential and steady growth, respectively, and Fis inhibits nucleoid condensation at logarithmic phase [33]. More than a half (230) of the Dps binding sites overlapped with Fis contact regions, and there were 62 genomic regions with an average length of 352 bp occupied by all four proteins considered here. Dps, therefore, shares its binding sites with at least three other structural proteins of the bacterial nucleoid.

### Sequences overrepresented in the Dps binding sites have a common motif with consensuses of other nucleoid proteins

The overlap of genomic regions occupied by Dps with binding sites of other nucleoid proteins makes the identification of a specific motif for Dps challenging and problematic. A preliminary search for over presented sequences was undertaken with MEME suite programs [54] for the 304 shortest samples from CS (total size—49789 bp, i.e. less than the 50000 bp limit of the software). Programs of this suite integrated into MEME-chip [67] were run in a discriminative mode with the 739 shortest sequences of UR (total size—49988 bp) and predicted three dominant motifs with E-values in the range of 4.6E-021–1.0E-011, including T(G/A)A(t/c)A, TA(c/t/g)(T/A), TT(T/a)(g/a) and one long sequence: (t/c)(g/c)(t/c)AGGCC(g/t)GATAAG(g/a)CG, which, however, was found only in 10 samples. DMINDA [55] predicted more potential candidates as consensuses of grouped overrepresented sequences. However, many of them contained A/T-rich motifs typical for nucleoid proteins, H-NS (consensuses: (a/g)ATA(A/t)(t/a) [49] or (T/g/c)(C/g/a)G(A/t)T(A/T)a(A/t)(t/a)t [68]), Fis (tG-t(g/t)(a/g)tTTTTT(c/g/a)-Ca [49]), IHF ((t/c)-(A/T)—(g/c)(t/c)—(A/t)(T/a)(t/a)(t/a) [51]), or some other transcription factors. For instance, TGAT, is a part of sequences recognized by H-NS, Fis and also FNR (aaa-tTGAt-ta-(a/g)TCAAtta(a/t)t [46] or tTGAt—-aTCAa [50]). During exponential, growth FNR has a limited number of binding sites, though their overlap with regions occupied by Dps is 1.9- and 2.7-fold higher than expected by chance (data from [47] and [50], respectively; S3 Table for FNR-bound regions). We, therefore, removed 336 genomic regions bound by other proteins from CS and used the remaining 115 sites occupied by Dps (average size 207 bp) for sequence analysis. In this case, the best motif found by MEME suite was (t/c)GATA (E = 4.8e-007), while DMINDA generated six groups with longer consensuses corresponding to this sequence for at least four nucleotides. They included: TG(G/a)(T/**c**)**GAT** (E = 4.7e-025), C**TG**(**A**/G)(**T**/c)**A**A (E = 1.7e-011), **GATA**-CG (E = 3.6e-009), cgCC**TGAT**GC (E = 9.9e-009), C-GG**CGAT** (E = 3.2e-007) and **CGATa**-CG (E = 3.6e-006). When 84 of 115 sequences containing at least one of those motifs were again used to find overrepresented motifs in the Dps binding sites, the dominant sequences revealed by MEME appeared to be GATA (E = 1.0e-007), while DMINDA suggested TG-tGAT as the most statistically significant (E = 4.6e-018) motif. Thus, the presence of overrepresented motifs in the Dps-bound genomic regions can not be ignored and they may be important for complex formation. However, a lack of a typical DNA-binding domain in the Dps protein and presence of the revealed motifs in the sites specifically recognized by other nucleotide proteins and transcription factors, also allows a suggestion that Dps prefers to bind DNA near the regions occupied by other proteins.

### Deletion of Dps differentially affects gene expression

Almost 50% of Dps-binding sites are immersed in the coding sequence of genes. The others at least partially encompass promoters or/and terminators, where different repeating sequences act as components of binding modules for the transcription factors, or transcription stop signals (Page 3, S3 Table). These targets include 66 sites located between divergently transcribed genes and 33 sites in the spacers between convergent genes. The genome has an equal number of spacers separating divergent and convergent genes, so this bias toward the promoter regions may indicate the involvement of Dps in transcription regulation. Consistent with this interpretation, Dps targets strongly overlap with RNA polymerase (RNAP) binding sites (Fig 4) and of the two types of genomic domains with extensive protein occupancy, only those that are enriched with RNA polymerase molecules were preferentially occupied by Dps (Fig 4F). Those domains (EPODs, **E**xtensive **P**rotein **O**ccupancy **D**omains) were identified without the use of any specific antibody and were classified by the authors [48] into two functional categories: highly expressed (he) and transcriptionally silent (ts) EPODs. Excessive overlap with Dps contacts was obtained only for heEPODs (Fig 4A). We, therefore, tested the propensity of Dps to affect expression efficiency of 8 genes using the *dps*-null mutant.

**Fig 5:**
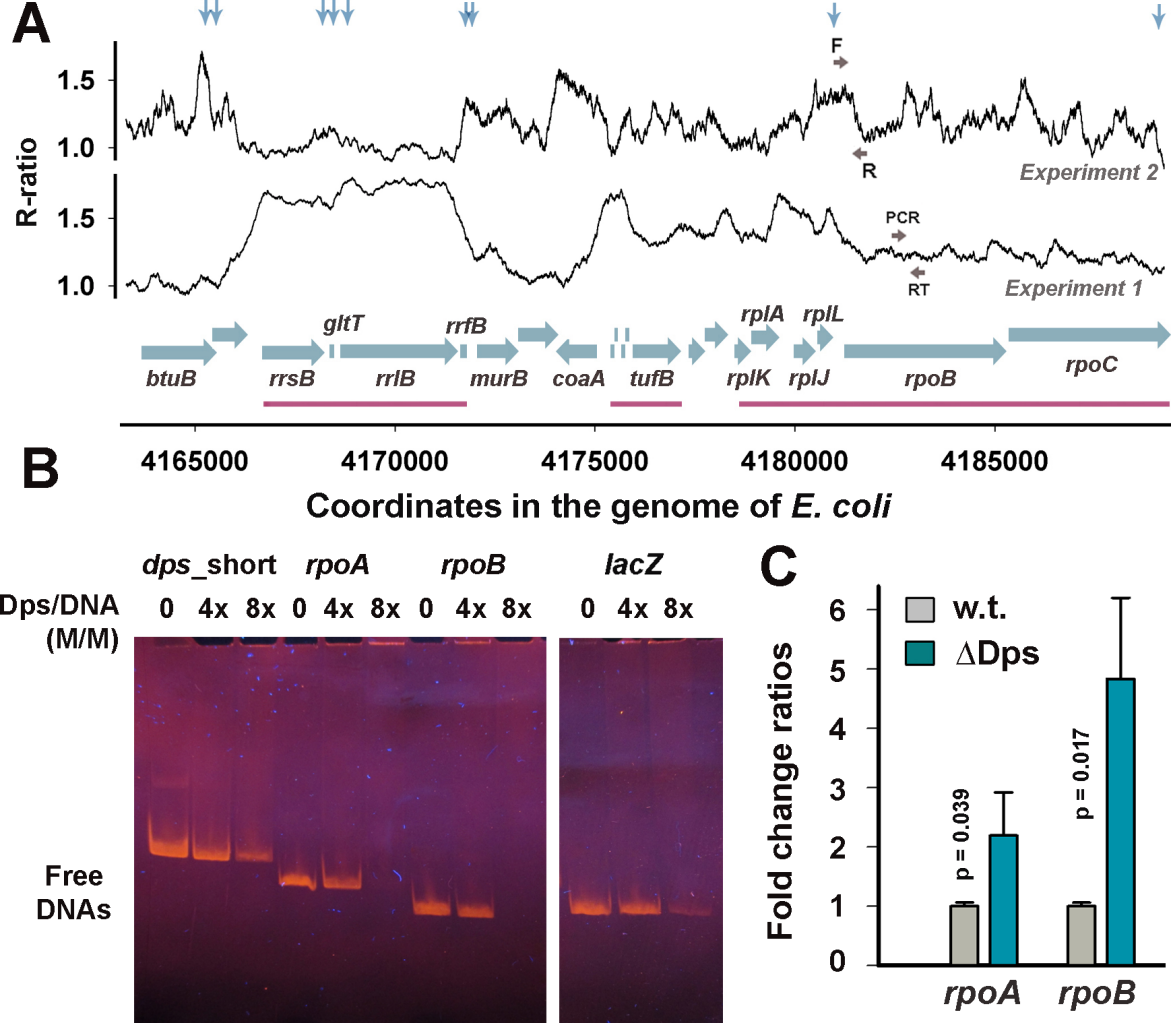
Deletion of the *dps* gene affects *rpoA* and *rpoB* expression. **A:** Profiles of the Dps binding sites obtained in two experiments (indicated) for the genomic region with three operons of ribosomal genes (running window of nine 35 bp bins). Genes are represented by blue horizontal arrows; magenta lines show ribosomal operons. Vertical arrows mark locations of inverted repeats (if longer than 7 bp). **B:** Band shift assays performed for indicated genomic loci. The regulatory region of the *dps* gene was used as a positive control for all band shift assays in this study. Fragment from the *lacZ* coding sequence was used as a reference gene for qRT-PCR experiments. Positioning of primers for amplification (**F** and **R**) is indicated in panel **A** of this figure, Fig 6A (for the *dps* regulatory region) and in S3 Fig (for *rpoA* and *lacZ*). **C:** Changes in the expression efficiency of selected genes in response to *dps* deletion. Primers used for reverse transcription and consecutive PCR are designated as RT and PCR, respectively, here and all other figures. Expression levels were estimated based on 3 and 5 biological samples (3–18 technical repeats in each) for *rpoA* and *rpoD*, respectively. Error bars show an average deviation. Statistical significance was assessed using Student’s t-test.

**Fig 6:**
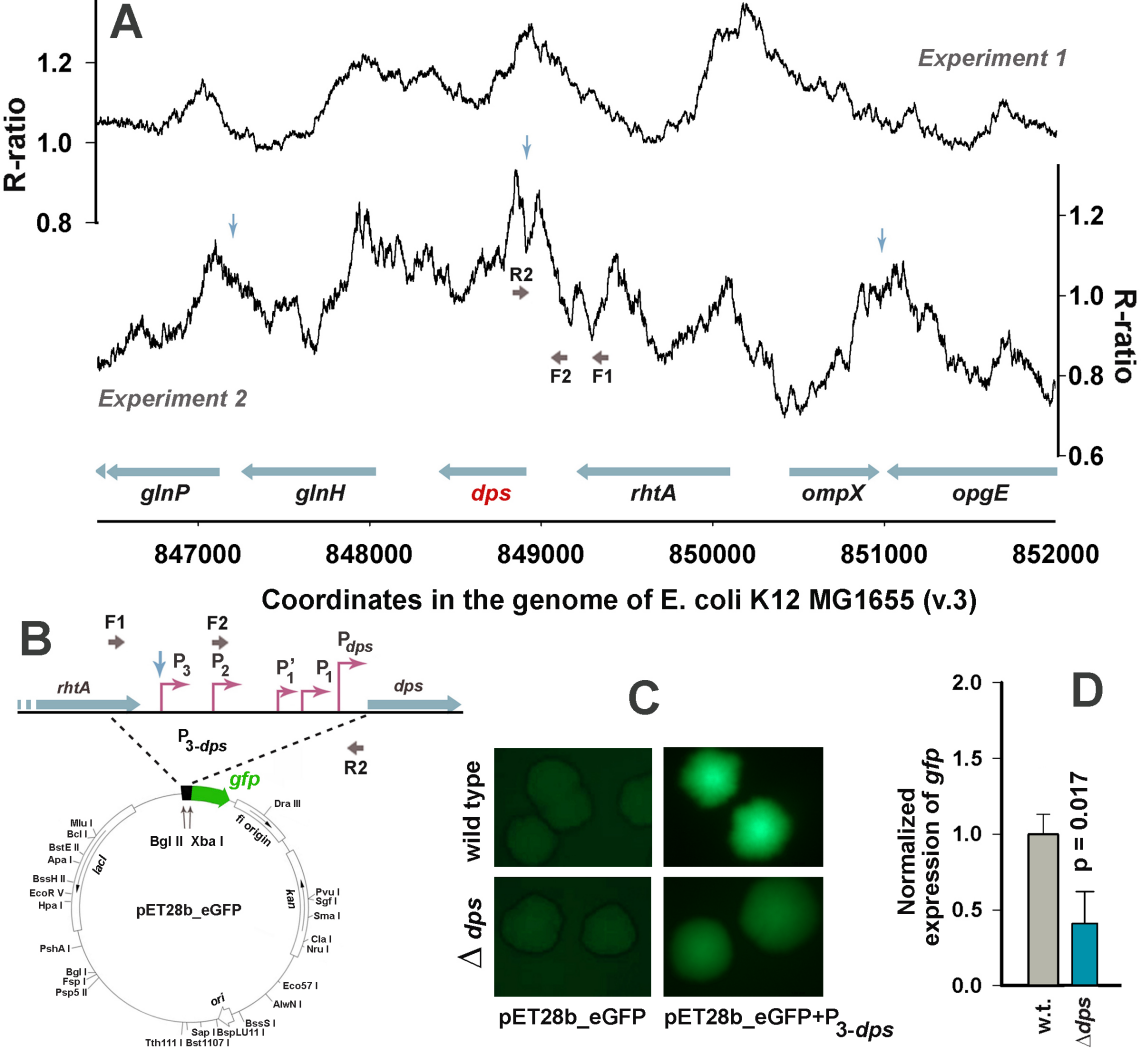
qRT-PCR and reporter assays revealed apparent positive autoregulation of the *dps* gene. **A:** Profiles of the Dps binding sites obtained in two experiments for the *dps* genomic locus (running window of nine 35 bp bins). Genes are displayed as blue horizontal arrows. Vertical arrows show locations of inverted repeats, if longer than 7 bp (blue). Primer pair F1-R2 was used for cloning the whole regulatory region in pET28b_eGFP. The *dps*_short fragment (positive control in all band-shift assays) was amplified with F2-R2. **B:** Scheme of the pET28b-eGFP reporter vector with the insert of the *dps* promoter region. **C:** Images of *E*. *coli* MG1655 cell colonies transformed by pET28b-eGFP plasmid with or without the promoter insert. Images were obtained by fluorescent microscope Leica with 2 or 0.5 sec exposition for control and experimental cells, respectively. **D:** Changes in the expression efficiency of *gfp* in response to *dps* deletion measured in qRT-PCR using *kan* as a reference gene. Expression levels were estimated based on 3 biological samples with 3 technical repeats in each. Error bars show an average deviation. Statistical significance was assessed using Student’s t-test.

Since the two ChIP-seq experiments revealed a fundamental difference in the interaction of Dps with ribosomal operons, it was reasonable to test the dependence of their expression on the presence of Dps. However, seven copies of rRNA genes complicate the data evaluation, so we took *rpoA* and *rpoB* genes, encoding α- and β–subunits of RNAP as representatives of genes from the operons of ribosomal proteins. Fig 5A shows the profiles of Dps contacts in the *rpoB* genomic region. There are three operons with ribosomal genes. All of them were fully occupied by Dps in the first experiment, including the part of the *rpoB* coding sequence, while in the second experiment the base line remained at the level of the control sample (**R** ≈ 1.0) and even fell below it within and nearby the *rrsB* operon. Almost the same was true for *rpoA*, transcribed as a polycistronic unit *rpsMKD*-*rpoA*-*rplQ* (S3A Fig). In the first experiment this and the neighboring *rplNXE-rpsNH-rplFR-rpsE-rpmD-rplO-secY-rpmJ* operon were extensively occupied by Dps (S3A Fig, Page 1 in S3 Table), while in the second assay the **R**-values only slightly exceeded the control level, and were below it in the *rpoA* region (S3B Fig). This remodeling was also detected for some other genes with growth-dependent expression. For instance, the *oppABCDF* operon (S4A Fig), encoding inner membrane proteins of ABC transporter and following stringent control reprogramming similar to ribosomal genes [69], was covered by Dps in the first experiment, while in the second one only two small peaks have maxima exceeding **R** = 1 (S4A Fig). In contrast, the binding of Dps to the *fis* genomic locus was detected only in the second experiment (S5A Fig). The *rho* gene, which encodes a transcription terminator and is expressed independent from ribosomal genes together with its neighborhood (S6A Fig), as well as the *dps* gene, selected as potential target for autoregulation (Fig 6A), represent examples of the genomic loci bound by Dps in both experiments, while the *lacZYA*, *mhpR-lacI* and *mhpABCDFE* operons exemplify regions remained unbound in both experiments (S3B Fig).

Demonstrating all possible types of similarity or difference between two experiments, the selected profiles confirmed certain correlation of detected peaks with disposition of long inverted repeats and also revealed some periodicity in the pattern of Dps binding. Quasi-regular alternation of the Dps-occupied and free DNA segments can be a specific feature in the mode of its interaction with the genome. Like nucleosomal fazing, this may be important for maintaining optimal conformation of active chromatin and for its rapid remodeling under stress conditions.

Band-shift assays were performed to characterize the ability of Dps to interact with linear DNA fragments representing the Dps-bound (Figs 5B, S4B, S5B and S6B) and unbound (Figs 5 and S5) regions. In all experiments the previously characterized DNA fragment, amplified with primers F2 –R2 from the regulatory region of the *dps* gene (dps_short) [19], was used as a positive control. Interaction efficiency was estimated based on the amount of DNA remained unbound. As a result, we observed the ability of Dps to interact with DNA fragments taken from the regions bound by Dps *in vivo* (Figs 5, S4–S6) and also confirmed its different affinity to the linear DNA fragments [19], but we did not find obvious correlation with ChIP-seq data in the efficiency of binding. For instance, from the two sequences unbound *in vivo*, taken from *fis* regulatory region (fis_up) and the *lacZ* coding area, first remained unbound in vitro (S5B Fig), while the second interacted with Dps even stronger than the control fragment “*dps*_short” (Fig 5B, profile in S3 Fig). Moreover, the DNA fragment from *rho* genomic loci representing the peak well reproduced *in vivo*, demonstrated rather weak binding in vitro (S6B Fig). Most likely this low correlation between *in vivo* and *in vitro* experiments is due to the ability of Dps to form complexes with partially melted ends of linear fragments [19] that can mask other types of interaction, if any. On the other hand, Dps-DNA binding in the cell might be significantly dependent from other proteins of bacterial nucleoid.

Functional dependence on Dps was detected for 5 out of 7 genes tested by qRT-PCR using *dps*-null mutant (Figs 5C, S4C–S6C). As expected, removal of Dps had a similar effect on *rpoA* and *rpoB* (Fig 5C). However, the level of their expression varied, and for *rpoB* it was unchanged in the mutant strain in one of 5 biological replicates, which probably reflects observed unstable interaction of Dps with ribosomal operons (Fig 5A). The increase in the amount of *rpoA* and *rpoB* mRNAs assumes a negative impact of Dps on their expression, which probably can be extended to ribosomal genes as well. Response of *rho* was the opposite (S6C Fig). The proteins of transcriptional machinery are, therefore, under the differential control of growth-dependent regulatory networks and it is possible that direct or indirect Dps-mediated stimulation of the ρ-dependent transcription termination is an important step in the process of genome packaging.

The strongest inhibition was detected for *fis* (S5C Fig). This was quite unexpected, since a stimulatory rather than inhibitory effect of the *dps* deletion would have been more anticipated if one takes into account the opposite changes in the concentrations of Fis and Dps during the growth of bacteria [4]. The *fis* gene is transcribed together with the upstream *dusB*, and the promoter of the latter is strongly inhibited by Fis [70, 71]. If Dps interferes with Fis, then its deletion can strengthen Fis-mediated suppression, explaining the observed inhibitory effect by the relieved competition.

We did not detect any changes in the amount of *oppA* or *oppB* mRNAs, but Dps appeared to be required for expression of *oppD* (S4C Fig). Taken into account operonic organization of this locus, competition with other transcription factors in the region between unaffected *oppA*—*oppB* and suppressed *oppD* seemed unlikely. Though, the analysis of available data sets revealed extended Fis contacts within *oppBCD* (wavy lines in S4 Fig). Thus, the detected inhibitory response to *dps* deletion in this case can be also explained by competitive interactions with Fis.

To test the possibility of feedback regulation for *dps*, the region containing all experimentally verified promoters [72, 73] was incorporated into pET28b-eGFP in front of *gfp* (Fig 6B). Removal of *dps* decreased the fluorescence of cells transformed with a resulting plasmid 1.5 fold (from 211.1±21.8 to 140.5±16.7 relative units, Fig 6C). Approximately the same inhibitory response was registered by qRT-PCR (primers gfp_F and gfp_R, S1 Table) for exponentially growing cells (Fig 6D). Promoter region of *dps* has two sites of primary contacts with Dps revealed by DNAse I footprinting [19]. One of them (65–120 bp upstream of P_*dps*_) fully covers the binding site for activator IHF and is located nearby repressor Fis contact regions (92–104 bp and 19–33 bp from P_*dps*_, respectively [74]). Another one (216–281 bp from P_*dps*_) has a 30 bp inverted repeat in the center (indicated in Fig 6A and 6B). Relieved interference with Fis in the first binding site perfectly explains inhibitory effect of dps deletion (Fig 6D), while lost interaction with inverted repeat can break topological state of the promoter region. The *dps* regulatory region can, thus, be suggested as a model for further studies of the Dps ability to bind structural elements and interfere with other regulatory proteins.

## Discussion

During exponential growth bacterial cells contain at least 6,000 monomers of Dps [3, 75], which are distributed along the entire uncondensed genome [76]. Upon starvation, Dps co-crystallizes with DNA [30] by hypothetical mechanism that does not require any sequence or structural specificity. To understand if Dps has any binding preferences we, therefore, selected cells harvested at exponential rather than stationary phase, although the intracellular concentration of Dps increases dramatically during steady growth [3, 75]. Automatic evaluation by CLC WB revealed several thousand of Dps targets with a total length of about 1,100,000–1,500,000 bp (S2 Table), while the cumulative size of areas occupied by Dps provided by the Matcher-based approach was 90,000–300,000 bp (Pages 1 and 2, S3 Table). In the exponential growing culture there are 500–700 Dps dodecamers per cell. If 60 bp are sufficient for their interaction with DNA (estimated in the titration experiments made *in vitro* [18]), then only 30,000–42,000 bp can be occupied. If our estimate of ~120 bp (based on the maxima positioning in the size distribution curves (S2B Fig)) reflects the topology of natural binding in the cell, then in 60,000–84,000 bp of the genome can be covered by Dps. These ranges are significantly smaller, than the total size of binding sites revealed by both methods in both experiments. Therefore, to maintain functional integrity of the genome during exponential growth Dps most likely uses different combinations of binding sites in different cells.

At stationary phase there are 7000–15,000 Dps dodecamers per cells, which can occupy 420,000–900,000 bp or 840,000–1,800,000 bp if, respectively, 60 or 120 bp are required for interaction with a single Dps particle. This roughly corresponds to the total length of Dps targets found by CLC GW (1,100,000–1,500,000 bp), suggesting a possibility that they are used as primary anchor sites for the genome packaging. But the question about the size of a single binding site still remains open, because both 60 bp and 120 bp are much longer than the physical size of the Dps dodecamer, ~7 nm [5], and analyzing the available AFM images, we have not found convincing evidence of DNA wrapping on the protein globule.

The large number of contact sites found for Dps is not very surprising, since the nucleoid proteins Fis, IHF, H-NS and even transcription factor FNR (during anaerobic growth) also bind to many locations in the genome, especially in stress conditions (500,000–1,600,00 bp) [46, 47, 49, 51]. This ubiquity assumes a substantial overlap between the contact sites of different proteins, and the crucial role of nucleoid-associated proteins in FNR occupancy at many of its target sites has been discussed [50]. Quantitatively estimating the superposition of Dps contact sites with the targets of three nucleoid proteins, we found the maximum overlap with the sites occupied by Fis. This is also not surprising, as it is believed that condensing the genome during steady growth; Dps replaces Fis that maintains the optimal structural state of the nucleoid during exponential growth. Less expected was an overlap with the binding sites of H-NS, a histone-like protein functioning as a transcription factor, or a structural protein [77, 78]. Unlike other nucleoid proteins, H-NS has an exclusively high specificity to horizontally acquired genes [65, 66] and *promoter islands* often located nearby [43, 62, 64]. We previously found that deletion of *hns* significantly activated transcriptional activity of the *islands*, while deletion of *dps* caused a differential effect of approximately the same scale as found in this study [56]. Now it became clear that both proteins (Fig 4A for Dps) have a strong affinity to the *islands*, where their functional interplay can be used to keep expression of foreign genes at the optimal level.

Although the genome has a large number of potential Dps binding sites, their distribution, even at a large scale was not uniform (Fig 1). The largest occupancy was observed for approximately 800,000 bp of the genome, including the origin of replication and 6 of 7 rRNA operons, where the most significant differences between the two ChIP-seq experiments were detected. This bias can be only partially explained by the gradient in the DNA copy number, since using R-ratio between read counts detected in the anti-Dps immunoprecipitated sequence libraries and the control sets, we obtained increase, rather than decrease in this bias for CS (Fig 1B, dashed plot). Thus, it is likely that the presence of a large number of Dps molecules in the last quarter of the genome is biologically meaningful permitting fast remodeling under stress conditions by turning off expression of ribosomal genes or by affecting replication initiation.

Both experiments indicated an interaction of Dps within or nearby oriC (positions 3925744–3925975) (S2 Table), which is important as it is known that Dps can delay replication by direct interaction with DnaA [79]. The genomic regions bound by Dps in this area contain many repeated sequences, including three pairs of inverted heptanucleotides within the origin (underlined below). One pair overlaps with two binding sites for IciA (in bold): AGA**GATC****TGTTCTATT**GT**GATCTCT****TATTA****G (**position 3925757). This protein inhibits replication by blocking the DNA opening by DnaA. Four other sequences can form two alternative complementary pairs: AGGATCATTAAC**TGTGAATGAT**CGGTGATCCT, or GATCATTAAC**TGTG****AATGAT**C (position 3925844–46, respectively) overlapping the DnaA binding site (in bold). Thus, it is possible that inverted repeats, forming a 4-way junction create a platform for interaction with the Dps-DnaA submolecular complex.

Enrichment of the Dps targets with inverted repeats (Fig 3) supports a previous suggestion that they are important for binding [19]. A total of 380 peak regions (84%) from CS possess 5–7 bp inverted repeats (Page 3, S3 Table), while in the set of unbound sequences this percentage is only 54.8% (Page 4, S3 Table). The role of inverted repeats in Dps binding is also supported by some correlation between their positioning and the peaks in the Dps binding profiles (Figs 5A, 6A and S3–S6). However, a large number of inverted repeats in the genome limits the possibility of their use for predictive mapping.

For direct repeats, which have the same large-scale profile as inverted repeats (Fig 1C), this ubiquity is even higher, and may explain why CS and UR differ in their presence only for long repeats (Fig 3). Almost all Dps contact regions (94%, Page 3, S3 Table) have tandem repeats, but they are also found in 78.6% of unbound sequences (Pages 4, S3 Table). For all that, one of the Dps primary contacts in the promoter region of its own gene covers the cluster with 5 bp direct repeats [19]. The ability of tandem repeats to facilitate or stabilize interaction with Dps is, therefore, unclear.

A similar conclusion can be reached regarding the role of sequence motifs over-represented in Dps binding sites GATA and TG-tGAT. Their presence can not be ignored, and direct interaction with Dps deserves special study by methods intended for this purpose. Currently it is likely that Dps can bind DNA near the regions occupied by other proteins. Then, revealed sequence motifs may belong to their specific targets. REP-elements can also contribute to the detected enrichment. Providing a platform for the structure-specific interaction they also “contaminate” bound regions with overrepresented motifs (consensus: GCC(g/**t****)****GAT**G-CG(a/g)CG(t/c)——(g/a)CG(c/t)CTT**ATC**(c/**a**)GGCCTAC, inverted repeats are underlined).

The fact that 6 of 8 genes with tested expression efficiency responded to the removal of *dps*, suggests that Dps can function as a transcription factor. This feature has not been previously described for this protein, though in many cases a “regulatory” effect can still be explained by changed availability of the promoter regions for transcriptional activators and/or inhibitors. Even specifically reduced amount of mRNA transcribed from intraoperonic *oppD* can be explained by the canceled competition for Fis in the *dps*-null mutant (S4 Fig). Whatever the mechanism of registered changes is, it became clear that variations in the intracellular Dps concentration may modulate the profile of mRNAs transcribed in exponentially growing cells. By now it has only been detected for the profiles of cellular proteins upon starvation [1].

The transcriptional activity of the *dps* promoters appeared to be also suppressed in the *dps*-null mutant (Fig 6D). Even though this suppression is most probably mediated by relieved interference with any of three regulatory proteins—Fis, H-NS and MntR-Mn^2+^ able to repress the dps promoter activity [80, 81], the data obtained indicate positive dependence of the *dps* expression on the cellular concentration of its protein product. Reporter assays revealed the same dependence for steady growing bacteria in cell colonies (Fig 6C). They strongly support the qRT-PCR data, though do not indicate that positive feedback also takes place during steady growth, because registered fluorescence can arise from GFP accommodated in cell colonies before they reached the stationary phase. It is clear, that positive autoregulation, either direct or competition-mediated, is favorable for the rapid genome packaging in case of emergency, but some cellular components are required to stop it when necessary and to remove Dps from the condensed chromosome. Some nutrients can fulfill this role during the exit from starvation. We recently found, for instance, that D-galacturonate and D-glucuronate, but not glucose, are capable of altering the oligomeric form of Dps [82], which may be crucial for remodeling. In any case, it became clear that interacting with genomic DNA Dps can affect expression of some genes. In most cases, including apparent positive autoregulation, this influence can be attributed to Dps competition with Fis. Functional interplay of these two proteins, which share common binding sites (Fig 4) and substitute each other depending on the environmental conditions, on promoters of genes from Fis regulon became thus of particular interest.

## Supporting information

S1 Fig Dps binds genomic DNA in a non-random manner. **A:** Correlation between the number of reads from immunoprecipitated and control libraries calculated for 100 bp bins (second experiment). All points, corresponding to rRNAs operons are marked in red. Blue dots correspond to the regions containing bins with **R**>1.4 in the first experiment (Fig 2A). Dashed line shows bisectrix of the plot. **B:** Distribution of the Dps binding sites the *E*. *coli* MG1655 genome. Two outer circles represent the gene map of the top and the bottom strands of the *E*. *coli* MG1655 genome. The red ticks on the third circle mark positions of rRNA operons. The profile of **R**-values and the distribution of reads registered in the control library were calculated for a 25 bp running window and plotted on the fourth and the fifth circles, respectively.
(TIF)

S2 Fig Typical Dps-binding sites are 100–130 bp in size. Histograms demonstrating the size-dependent distribution of the Dps-binding sites, revealed in two ChIP-seq experiments by CLC GW (**A**) and the Matcher (**B**) (blue and green plots, respectively). Solid and dashed lines correspond to the peaks found with default and stringent settings, respectively. The magenta plot in panel **B** corresponds to CS.
(TIF)

S3 Fig Dps reproducibly binds to the operons of ribosomal proteins, leaving the *lacZYA*, *mhpR-lacI* and *mhpABCDFE* operons unbound. Profiles of the Dps binding sites obtained in the two experiments (indicated) for the genomic region containing two operons of genes encoding ribosomal proteins (**A**) and operons of metabolic genes (**B**) (running window of nine 35 bp bins). Genes are shown by blue horizontal arrows, operons are indicated by magenta lines. Vertical arrows mark locations of inverted repeats (if longer than 7 bp). Positioning of primers used for amplification (F and R) and qRT-PCR (RT and PCR) are indicated.
(TIF)

S4 Fig Deletion of *dps* affected expression of *oppD* without any impact on *oppA* and *oppB*. **A:** Profiles of the Dps binding sites obtained in the two experiments (indicated) for the genomic region associated with the *oppABCDF* operon (running window of nine 35 bp bins). Disposition of genes is indicated by blue horizontal arrows. Genes transcribed as a polycistronic unit are underlined by magenta line. Vertical arrows mark locations of inverted repeats (if longer than 7 bp). Wavy lines indicate sites bound by H-NS, Fis and IHF in the data sets obtained in [46, 47, 49–51] and used for intersection analysis (Fig 4). Positioning of primers used for amplification (F and R) and qRT-PCR (RT and PCR) are indicated. **B:** Band shift assays performed for indicated DNA fragments. **C:** Dependence of expression efficiency in response to *dps* deletion. Expression levels were estimated based on 5, 7 and 6 biological samples (3 technical repeats in each) for *oppA*, *oppB* and *oppD*, respectively. Error bars show an average deviation. Statistical significance was assessed using Student’s t-test.
(TIF)

S5 Fig Deletion of *dps* significantly affects *fis* expression. **A:** Profiles of the Dps binding sites obtained in the two experiments (indicated) for the genomic region associated with the *dusB*-*fis* operon (running window of nine 35 bp bins). Disposition of genes is indicated by blue horizontal arrows. Genes transcribed as a polycistronic unit are underlined by magenta line. Vertical arrows mark locations of inverted repeats (if longer than 7 bp). Positioning of primers used for amplification (F and R) and qRT-PCR (RT and PCR) are indicated. **B:** Band shift assays, performed for indicated DNA fragments. **C:** Dependence of expression efficiency of *fis* in response to *dps* deletion. Expression levels were estimated based on 4 biological samples with 3 technical repeats in each. Error bars show an average deviation. Statistical significance was assessed using Student’s t-test.
(TIF)

S6 Fig Deletion of *dps* significantly affects expression of the *rho* gene. **A:** Profiles of the Dps binding sites obtained in two experiments (indicated) for the genomic region associated with the *rho* gene. Disposition of genes is indicated by blue horizontal arrows. Genes transcribed as a polycistronic unit are underlined by magenta line. Vertical arrows mark locations of inverted repeats (if longer than 7 bp). Positioning of primers used for amplification (F and R) and qRT-PCR (RT and PCR) are indicated. **B:** Band-shift assays, performed for the indicated DNA fragments. **C:** Dependence of expression efficiency of *rho* in response to *dps* deletion. Expression levels were estimated based on 3 biological samples with 6 technical repeats in each. Error bars show an average deviation. Statistical significance was assessed using Student’s t-test.
(TIF)

S1 Table Primers used in the study. (DOC)

S2 Table Peak regions found by CLC GW in both experiment using different settings. (XLSX)

S3 Table Dps-bound and unbound regions found by the Matcher-based approach. (XLSX)
