# Supplementary figures and images for: The nucleoid protein Dps binds genomic DNA of Escherichia coli in a non-random manner
Source: PLoS One. 2017 Aug 11;12(8):e0182800. doi: 10.1371/journal.pone.0182800 (PMC5553809; doi:10.1371/journal.pone.0182800)

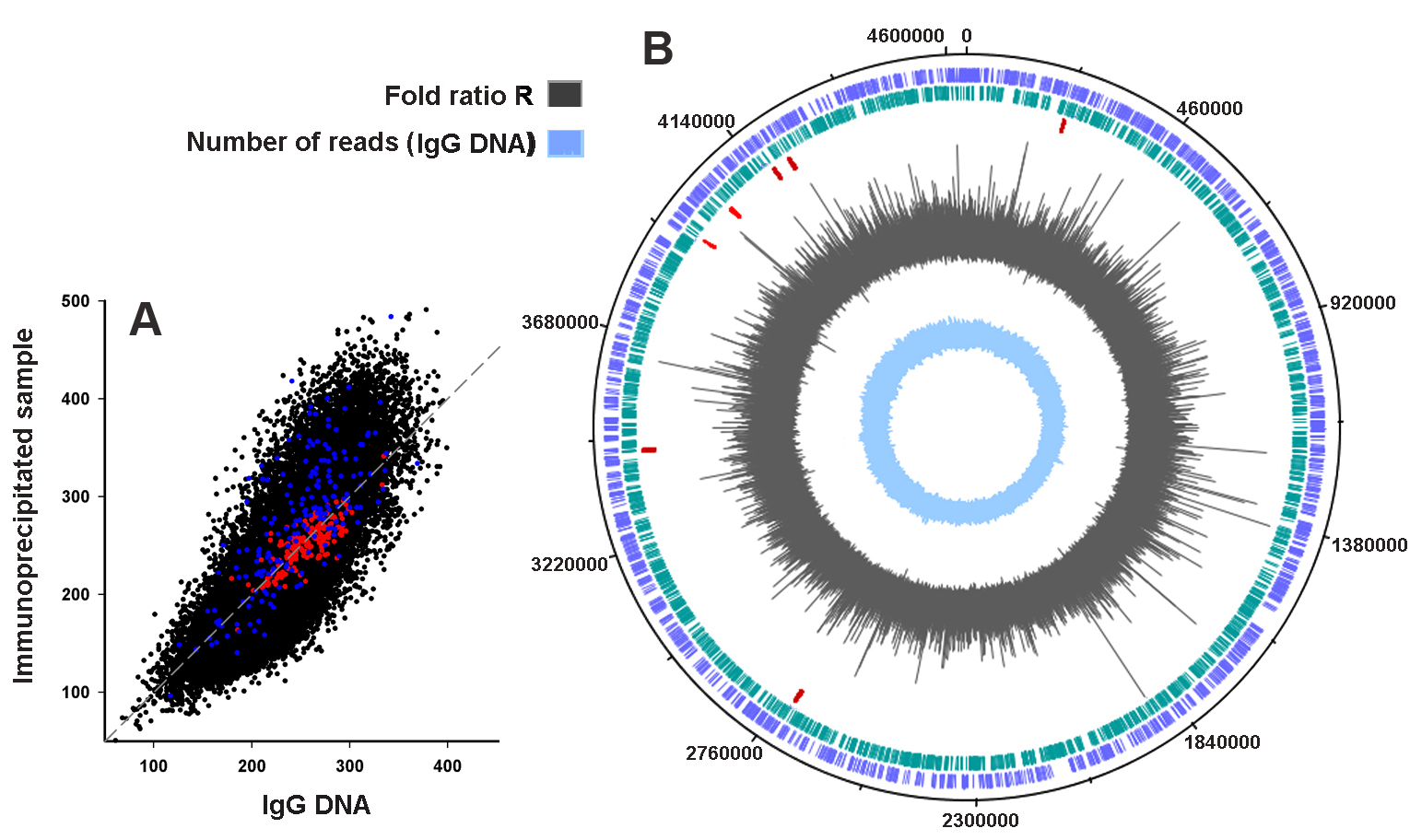

Supplement: S1 Fig — A: Correlation between the number of reads from immunoprecipitated and control libraries calculated for 100 bp bins (second experiment). All points, corresponding to rRNAs operons are marked in red. Blue dots correspond to the regions containing bins with R>1.4 in the first experiment (Fig 2A). Dashed line shows bisectrix of the plot. B: Distribution of the Dps binding sites the E. coli MG1655 genome. Two outer circles represent the gene map of the top and the bottom strands of the E. coli MG1655 genome. The red ticks on the third circle mark positions of rRNA operons. The profile of R-values and the distribution of reads registered in the control library were calculated for a 25 bp running window and plotted on the fourth and the fifth circles, respectively. (TIF) [file pone.0182800.s001.tif]

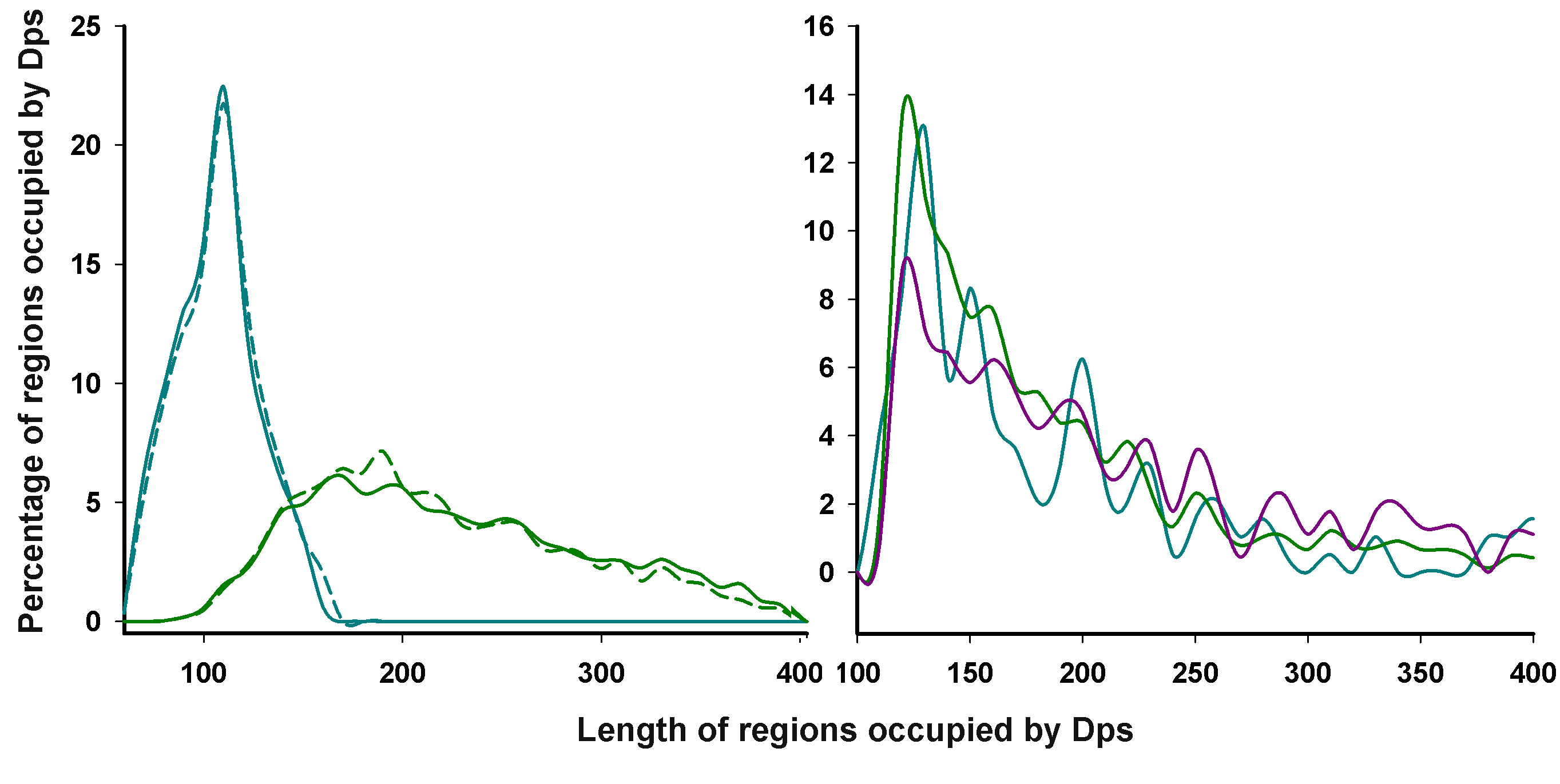

Supplement: S2 Fig — Histograms demonstrating the size-dependent distribution of the Dps-binding sites, revealed in two ChIP-seq experiments by CLC GW (A) and the Matcher (B) (blue and green plots, respectively). Solid and dashed lines correspond to the peaks found with default and stringent settings, respectively. The magenta plot in panel B corresponds to CS. (TIF) [file pone.0182800.s002.tif]

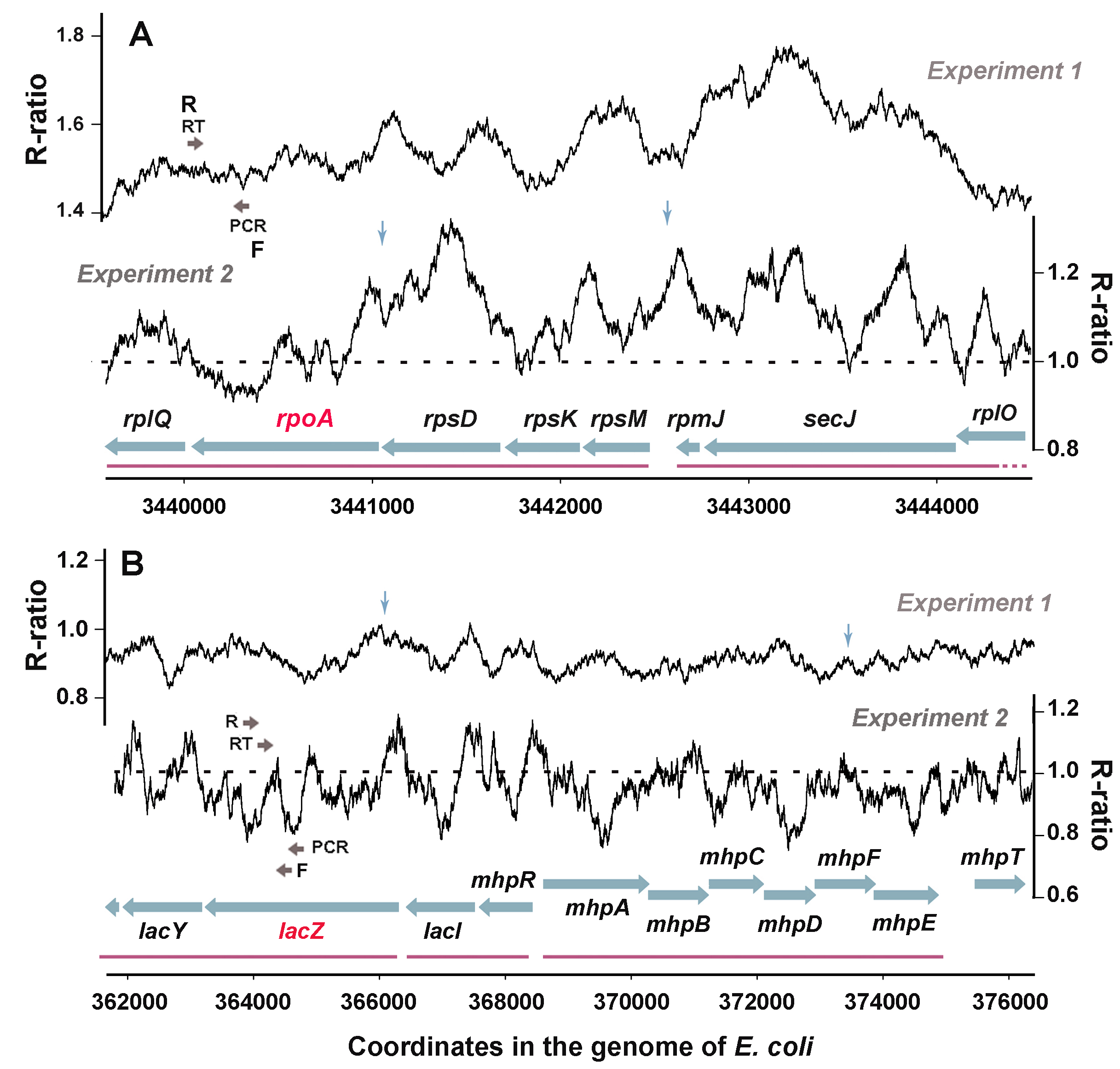

Supplement: S3 Fig — Profiles of the Dps binding sites obtained in the two experiments (indicated) for the genomic region containing two operons of genes encoding ribosomal proteins (A) and operons of metabolic genes (B) (running window of nine 35 bp bins). Genes are shown by blue horizontal arrows, operons are indicated by magenta lines. Vertical arrows mark locations of inverted repeats (if longer than 7 bp). Positioning of primers used for amplification (F and R) and qRT-PCR (RT and PCR) are indicated. (TIF) [file pone.0182800.s003.tif]

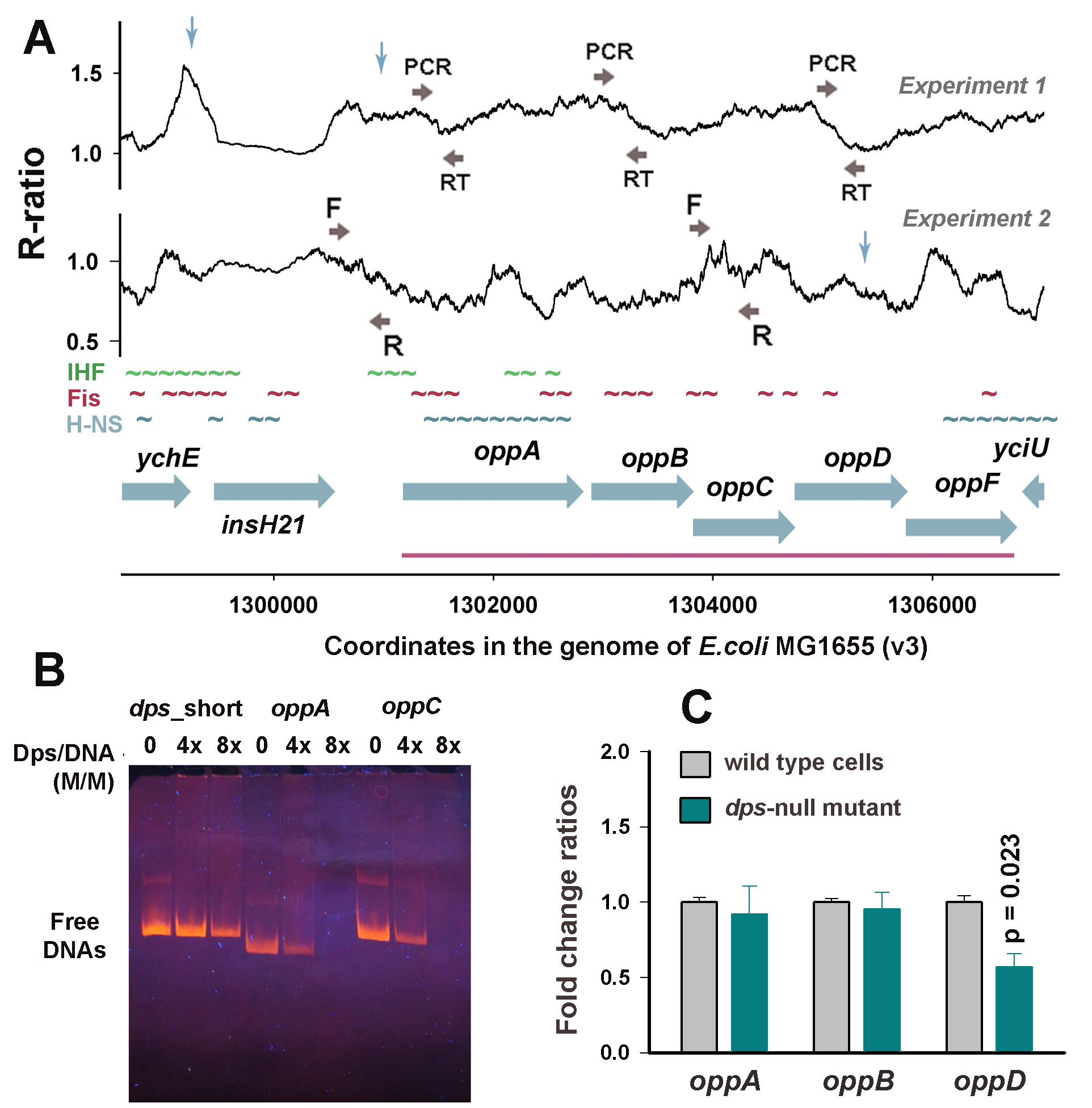

Supplement: S4 Fig — A: Profiles of the Dps binding sites obtained in the two experiments (indicated) for the genomic region associated with the oppABCDF operon (running window of nine 35 bp bins). Disposition of genes is indicated by blue horizontal arrows. Genes transcribed as a polycistronic unit are underlined by magenta line. Vertical arrows mark locations of inverted repeats (if longer than 7 bp). Wavy lines indicate sites bound by H-NS, Fis and IHF in the data sets obtained in [46, 47, 49–51] and used for intersection analysis (Fig 4). Positioning of primers used for amplification (F and R) and qRT-PCR (RT and PCR) are indicated. B: Band shift assays performed for indicated DNA fragments. C: Dependence of expression efficiency in response to dps deletion. Expression levels were estimated based on 5, 7 and 6 biological samples (3 technical repeats in each) for oppA, oppB and oppD, respectively. Error bars show an average deviation. Statistical significance was assessed using Student’s t-test. (TIF) [file pone.0182800.s004.tif]

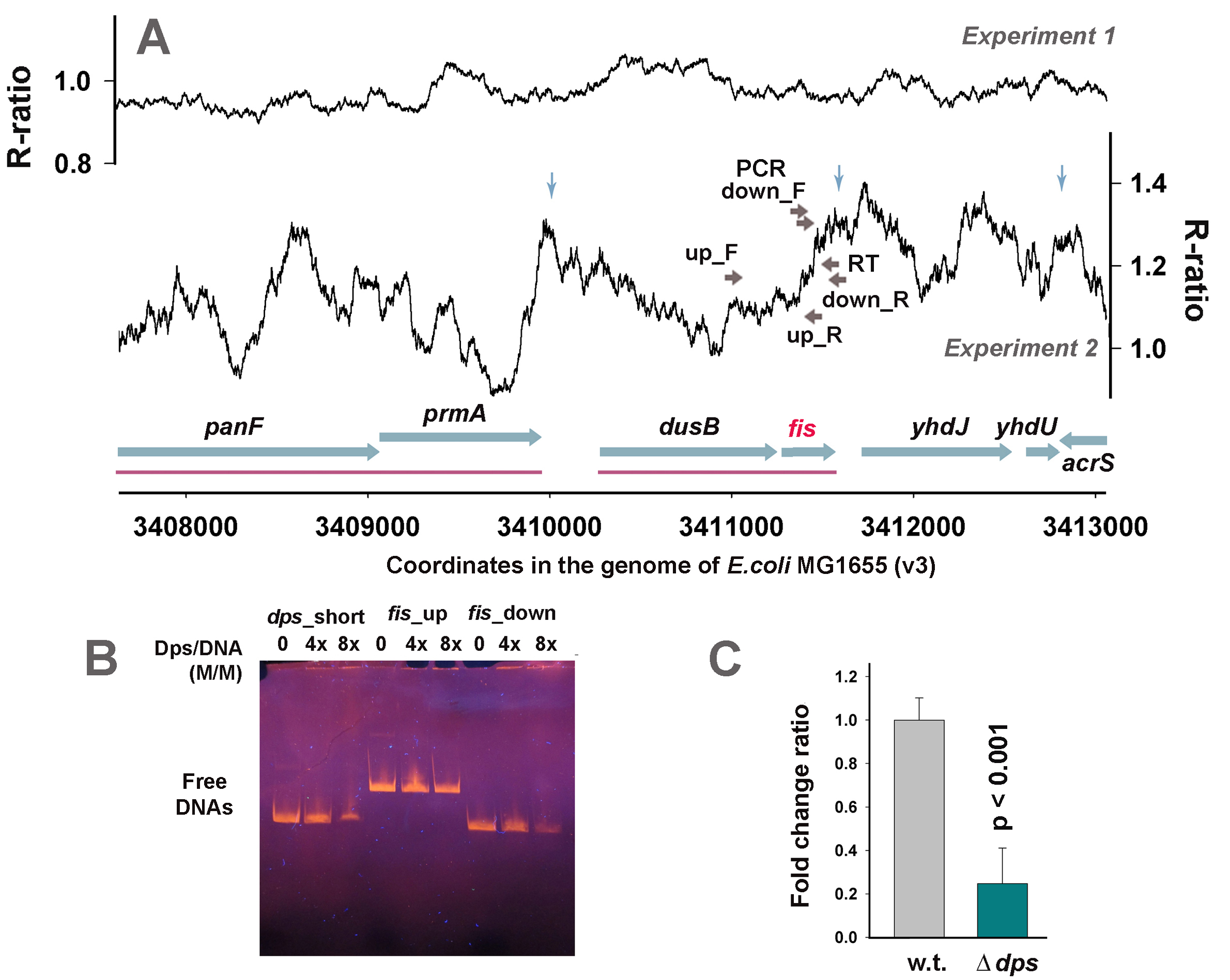

Supplement: S5 Fig — A: Profiles of the Dps binding sites obtained in the two experiments (indicated) for the genomic region associated with the dusB-fis operon (running window of nine 35 bp bins). Disposition of genes is indicated by blue horizontal arrows. Genes transcribed as a polycistronic unit are underlined by magenta line. Vertical arrows mark locations of inverted repeats (if longer than 7 bp). Positioning of primers used for amplification (F and R) and qRT-PCR (RT and PCR) are indicated. B: Band shift assays, performed for indicated DNA fragments. C: Dependence of expression efficiency of fis in response to dps deletion. Expression levels were estimated based on 4 biological samples with 3 technical repeats in each. Error bars show an average deviation. Statistical significance was assessed using Student’s t-test. (TIF) [file pone.0182800.s005.tif]

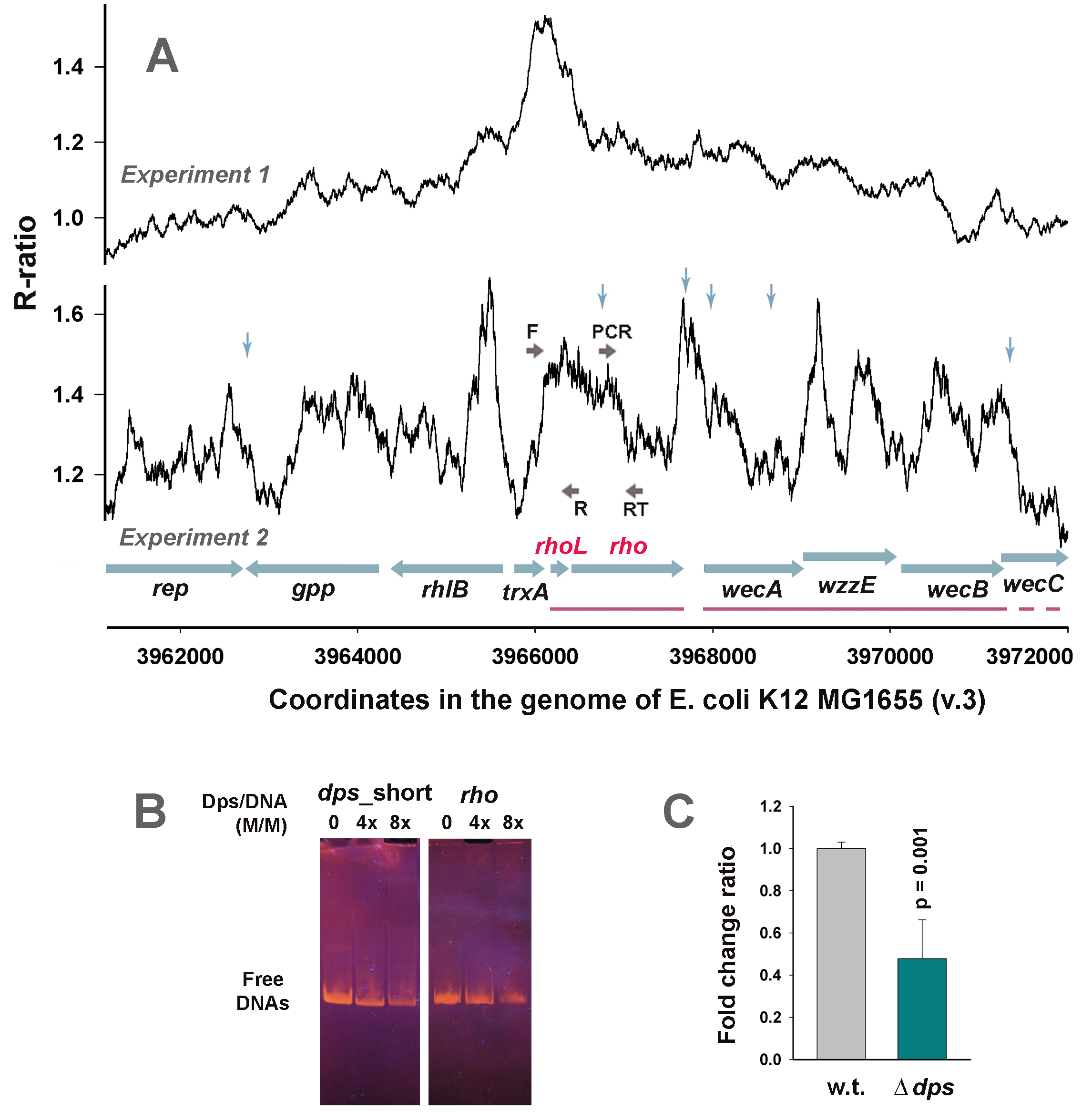

Supplement: S6 Fig — A: Profiles of the Dps binding sites obtained in two experiments (indicated) for the genomic region associated with the rho gene. Disposition of genes is indicated by blue horizontal arrows. Genes transcribed as a polycistronic unit are underlined by magenta line. Vertical arrows mark locations of inverted repeats (if longer than 7 bp). Positioning of primers used for amplification (F and R) and qRT-PCR (RT and PCR) are indicated. B: Band-shift assays, performed for the indicated DNA fragments. C: Dependence of expression efficiency of rho in response to dps deletion. Expression levels were estimated based on 3 biological samples with 6 technical repeats in each. Error bars show an average deviation. Statistical significance was assessed using Student’s t-test. (TIF) [file pone.0182800.s006.tif]
