# Supplementary material for: The nucleoid protein Dps binds genomic DNA of Escherichia coli in a non-random manner
Source: PLoS One. 2017 Aug 11;12(8):e0182800. doi: 10.1371/journal.pone.0182800 (PMC5553809; doi:10.1371/journal.pone.0182800)
Supplement: S1 Table — (DOC) [file pone.0182800.s007.doc]

**Table S1 Primers used in the study**

| Name | Sequence |
| --- | --- |
| dps_del_1 | 5’-TCGTTAATTACTGGGACATAACATCAAGAGGATATGAAATTATG ATTCCGGGGATCCGTCGAC-3’ |
| dps_del_2 | 5’-TCGGGTACTAAAGTTCTGCACCATCAGCGATGGATTTATTCGAT GTAGGCTGGAGCTGCTTC-3’ |
| dps_cl_1 | 5’- TAATTTCTAGAACATAACATCAAGAGG-3’ |
| dps_cl_2 | 5’- AGCTCTAGATTTATTCGATGTTAG-3’ |
| dps_F1 | 5’-GGAAGATCTTCCTCGGAGAAACACT-3’ |
| dps_F2 | 5’-ATGCAGATCTTCTCGCTACTTTTC-3’ |
| dps_R2 | 5’-TCCTCTAGATGTTATGTCCCAGT-3’ |
| fis_up_F | 5’-TTTGGCAGAGGTTAAGCGCT-3’ |
| fis_up_R | 5’-GTACCAGCTCATAGAGGTCA-3’ |
| fis_down_F | 5’-GACCTCTATGAGCTGGTACT-3’ |
| fis_down_R | 5’-TCACTCCCTTTGTGACACCT-3’ |
| fis_RT | 5’-GCATCACCATGTCCAACAGG-3’ |
| fis_PCR | 5’- CCAAAAACCCCTGCGTGACTC-3’ |
| rpoA_RT(F) | 5’-CATGCCCAGAGACAGTCCAC-3’ |
| rpoA_PCR(R) | 5’-CCAGAGTTCGATCCGATCCT-3’ |
| rpoB_F | 5’-CCAAGGCGGTAGCTGACGTATT-3’ |
| rpoB_R | 5’-CACAAGTTCTGGATGTACCTTA-3’ |
| rpoB_RT | 5’-TTGTGCGTAATCTCAGACAG-3’ |
| rpoB_PCR | 5’-CTCTGGGCGATCTGGATACC-3’ |
| rho_F | GAAAGAGTTCCTCGACGCTA |
| rho_R | GTCACAGGAGAGTACGCAGA |
| rho_RT | 5’-TTCGTCGATCAGCAGAACCATC-3’ |
| rho_PCR | 5’-TAACTGCTCGCGTACTGGATC-3’ |
| oppA_F | **5’-TCCCCGCTTATTCGCACC-3’** |
| oppA_R | 5’-ACTCGCCAATACATTTTGC-3’ |
| oppA_RT | 5’-TCGGATCAACAGAACGTTGC-3’ |
| oppA_PCR | 5’-GATCTTGACGGTCATCCAGC-3’ |
| oppB_RT | 5’-CCGCAAGGAAAAATGCTGCG-3’ |
| oppB_PCR | 5’-TGACACAGTATTTCAGCTACC-3’ |
| oppD_F | 5’**-GGTTGCCAGTCTGATAGT-3’** |
| oppD_R | 5’**-CATCATTACCGAATCCAC-3’** |
| oppD_RT | 5’-GCATCCGCACCGACTCTTCA-3’ |
| oppD_PCR | 5’-CCCAATGACTTCGTTGAATC-3’ |
| lacZ_RT | 5’-CCATCCAGTGCAGGAGCTCG-3’ |
| lacZ_PCR | 5’-**CAACCCGTGGTCGGCTTACG-3’** |
| lacZ_F | CGGTGAAGTGCCTCTGGATG |
| lacZ_R1 | CTGGTGGTCAGATGCGGGAT |
| lacZ_R2 | GGGAGCGTCACACTGAG |
| gfp_PCR | 5’-TTGTACTCCAGCTTGTGCCC-3’ |
| gfp_RT | 5’-ACGACGGCAACTACAAGACC-3’ |
| kan_PCR | 5’-TCAACGGGAAACGTCTTGCT-3’ |
| kan_RT | 5’-TCGGGCTTCCCATACAATCG-3’ |
